# pH-responsive Oral liposomal delivery of hydrogen sulfide donor GYY4137 enables colon-targeted therapy for inflammatory bowel disease

**DOI:** 10.1186/s12951-025-03753-9

**Published:** 2025-11-11

**Authors:** Chiwoo Oh, Jee-Eun Hwang, Hyunjoon Yim, Haena Park, Seo-young Kim, Jae Kyoo Lee, Hyung-Jun Im

**Affiliations:** 1 Department of Applied Bioengineering, Graduates School of Convergence Science and Technology, Seoul National University, Seoul, Republic of Korea; 2 Department of Molecular Medicine and Biopharmaceutical Sciences, Graduate School of Convergence Science and Technology, Seoul National University, Seoul, Republic of Korea; 3 Cancer Research Institute, Seoul National University, Seoul, 03080 Republic of Korea; 4 Research Institute for Convergence Science, Seoul National University, Seoul, 08826 Republic of Korea; 5 Precision Vaccines Program, Department of Pediatrics, Boston Children’s Hospital, Boston, MA USA; 6 Harvard Medical School, Boston, MA USA

**Keywords:** Inflammatory bowel disease (IBD), H_2_S, Oral delivery, PH-responsive colon targeted delivery, Nanomedicine

## Abstract

**Background:**

Although therapeutic options for inflammatory bowel disease (IBD) have advanced, many patients still experience suboptimal clinical response, systemic side effects, or difficulty maintaining long-term treatment adherence. Hydrogen sulfide (H₂S), an endogenous gasotransmitter with potent anti-inflammatory and mucosal-protective properties, has shown promise as a therapeutic agent for IBD. However, clinical application has been constrained by its instability in low pH and the need for parenteral administration.

**Results:**

Here, we report the first orally administered, pH-responsive liposomal formulation of the H₂S donor GYY4137, specifically designed for colon-targeted delivery. This system, termed oral hydrogen sulfide donor-loaded liposome (Oral H₂S lipo), employs a pH-sensitive Eudragit S100 coating that forms protective aggregates under acidic gastric conditions (pH 2) to shield the liposomes and suppress premature H₂S release. In this environment, Oral H₂S lipo limited cumulative release of H_2_S to 12.13% over 8 days, representing a ~ fivefold reduction compared to free GYY4137 (60% release). Upon exposure to colonic pH (≥ 7), the coating dissolved, restoring the native liposomal state as evidenced by a reduction in polydispersity index (PDI) from 0.777 to 0.076, and enabling sustained H_2_S release. The formulation also exhibited high drug loading efficiency (74.65%), stable physicochemical properties across gastrointestinal pH conditions for at least 14 days and excellent in vitro biocompatibility over a wide concentration range (0–120 μM). In vivo fluorescence imaging in a dextran sodium sulfate (DSS)-induced colitis model demonstrated that DiR-labeled Oral H₂S lipo achieved ~ 2.4-fold higher colonic accumulation than free DiR (p < 0.01) and ~ 1.7-fold higher than DiR-H₂S lipo (p < 0.05), validating the functionality of the pH-responsive coating for site-specific drug release. Therapeutic studies further showed improved colon length, reduced histological inflammation, and preservation of mucosal structure.

**Conclusions:**

These findings demonstrate that Oral H₂S lipo enables effective, site-specific delivery of H₂S to inflamed colonic tissue, offering a clinically relevant platform to overcome limitations of conventional H₂S donor therapies in IBD management.

**Graphical abstract:**

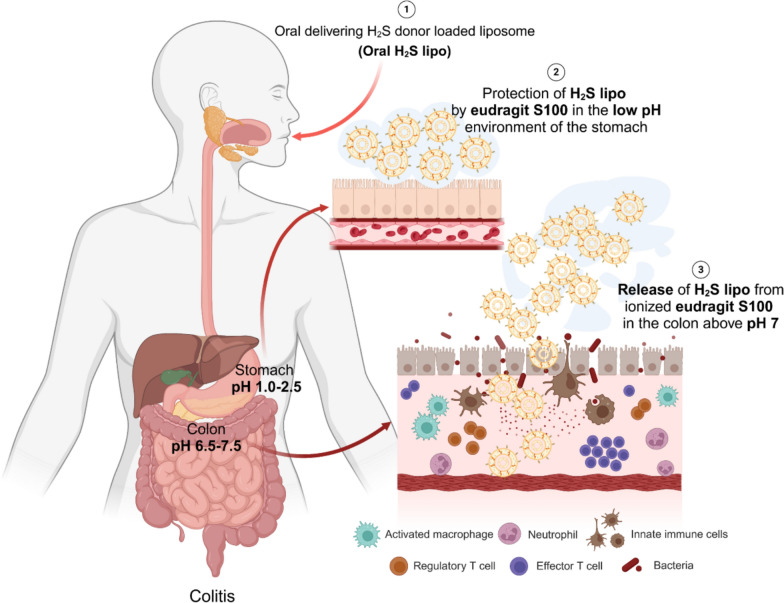

**Supplementary Information:**

The online version contains supplementary material available at 10.1186/s12951-025-03753-9.

## Background

Inflammatory bowel disease (IBD), encompassing primarily ulcerative colitis (UC) and Crohn’s disease, refers to inflammatory disorders affecting the gastrointestinal (GI) tract [1, 2]. As of 2021, the annual incidence of IBD reached ~ 375,000 cases globally, representing a substantial increase from 1990, with the global age-standardized incidence rate (ASIR) also rising over the same period [3, 4]. The incidence of IBD has been increasing not only in North America and Europe, but also in Asia, highlighting its growing global public health significance [5–7]. Among IBD subtypes, ulcerative colitis is characterized by chronic inflammation confined to the mucosal layer of the colon, typically beginning in the rectum and potentially progressing proximally to involve the entire colon [8, 9]. Clinical manifestations of UC commonly include abdominal pain, diarrhea, weight loss, fever, and hematochezia [10, 11]. Although the precise mechanism of UC remains incompletely elucidated, current evidence indicates a multifactorial etiology involving genetic susceptibility, environmental influences, disruption of gut microbial communities, and immune dysregulation [12, 13].

The treatment of IBD ranges from mild to severe cases, employing agents such as anti-inflammatory drugs (e.g., 5-aminosalicylic acid [5-ASA] and corticosteroids); biologic agents targeting tumor necrosis factor-alpha (TNF-α) and the interleukin (IL)−12/23 pathways; immunosuppressive drugs that inhibit the Janus kinase (JAK) pathway; and antibiotics [14–16]. Alternative strategies, including fecal microbiota transplantation (FMT), have also been explored [17]. However, response rates to current treatments vary widely, ranging from 16%–70%, and approximately 10–15% of patients with refractory disease ultimately require colectomy [18]. These limitations underscore the urgent need for more effective and targeted treatment approaches.

Hydrogen sulfide (H₂S) is an endogenous gasotransmitter involved in diverse physiological processes [19, 20]. Given its critical role in maintaining physiological homeostasis, abnormal fluctuations in H₂S concentrations have been linked to various pathological conditions, including diabetes [21], hypertension [22], atherosclerosis [23], and gastrointestinal diseases [24]. H_₂_S exhibits significant anti-inflammatory and cytoprotective properties within the gastrointestinal tract, making H₂S-releasing compounds attractive therapeutic candidates for the treatment of gastrointestinal disorders [25–27]. Previous research has shown promising therapeutic outcomes following the administration of H₂S donors in models of colitis and gastritis [25, 28–32]. Nonetheless, most earlier studies employed direct injection of H₂S donors, a route that poses major clinical limitations, including patient discomfort [33–37], difficulty in long-term administration, and inadequate control of release kinetics [38–41].

Nanoparticle-based oral drug delivery strategies have attracted increasing attention as promising approaches for the treatment of IBD [42–44]. Most existing IBD therapies rely on repeated intravenous administration, which offers rapid systemic delivery via the bloodstream but often causes off-target accumulation in healthy organs and systemic side effects [45–47]. By comparison, oral formulations result in slower systemic drug delivery but offer significant advantages for IBD therapy, including localized colon targeting, non-invasive administration, and greater patient compliance [48–50]. Nonetheless, oral drug delivery faces substantial challenges, such as reduced drug stability due to gastrointestinal physiological barriers, and difficulty in targeting specific inflamed regions of the colon [51, 52]. To enhance therapeutic outcomes in IBD, there is an urgent and unmet need to develop novel drug delivery systems that: (1) maintain the beneficial properties of oral administration, such as its non-invasive nature and high patient compliance; (2) minimize systemic toxicity by reducing off-target effects; (3) ensure drug stability; and (4) enable efficient and site-specific delivery to inflamed colonic tissues. We hypothesize that liposomes enhance the stability of encapsulated drugs and facilitate site-specific delivery to inflamed colonic tissues in IBD, thereby reducing systemic toxicity and improving therapeutic efficacy. Herein, we developed a novel orally administered, colon-targeted liposomal system—referred to as Oral H₂S lipo—for the H₂S donor GYY4137. To our knowledge, this is the first application of GYY4137 in an oral drug delivery system for IBD therapy. This strategy was designed to overcome both the inherent limitations of H₂S donors and the challenges of oral administration. The formulation was constructed by encapsulating GYY4137 within liposomes and coating the surface with Eudragit S100, a well-established pH-sensitive polymer that dissolves at pH ≥ 7 and is widely recognized for colon-targeted delivery due to its safety and biocompatibility [53]. To evaluate system functionality, we first confirmed that Eudragit S100 appropriately responds to pH changes, thereby enabling site-specific formulation release. Moreover, the formulation exhibited excellent structural integrity and drug-loading stability under various pH conditions that simulate the gastrointestinal environment, particularly under harsh acidic conditions. Furthermore, in vivo imaging using a colitis mouse model demonstrated that oral hydrogen sulfide donor-loaded liposome (Oral H₂S lipo) possesses superior colon-targeting capability. Collectively, these findings establish Oral H₂S lipo as the first oral formulation of GYY4137 for IBD therapy and underscore its potential as a novel therapeutic option for IBD.

## Methods

### Synthesis of H_2_S lipo

Uncoated hydrogen sulfide donor-loaded liposome (H₂S lipo) was prepared at a mass ratio of DSPC:DSPE-mPEG:cholesterol = 6:1:1. DSPC, DSPE-mPEG, and cholesterol were dissolved in a chloroform/methanol (2:1, v/v) mixture. To load GYY4137 (an H₂S donor) into the liposomes, 133.3 µM (265.6 mM) of GYY4137 was first dissolved in DMSO and then mixed with the previously prepared lipid solution. To remove the organic solvents from the mixture, nitrogen (N₂) purging was performed to evaporate them, followed by overnight vacuum drying to ensure complete removal of any residual solvents, resulting in the final lipid thin film. The obtained lipid film was then hydrated with triple-distilled water (3 × DW). To obtain uniform liposomes, the dispersion was sonicated using a tip sonicator (12 min, 24% amplitude). Finally, H₂S lipo was filtered through a 200 nm syringe filter and further purified using a PD-10 column (size-exclusion chromatography) to remove free GYY4137, yielding the final formulation.

### Synthesis of Oral H_2_S lipo

**Fig. 1:**
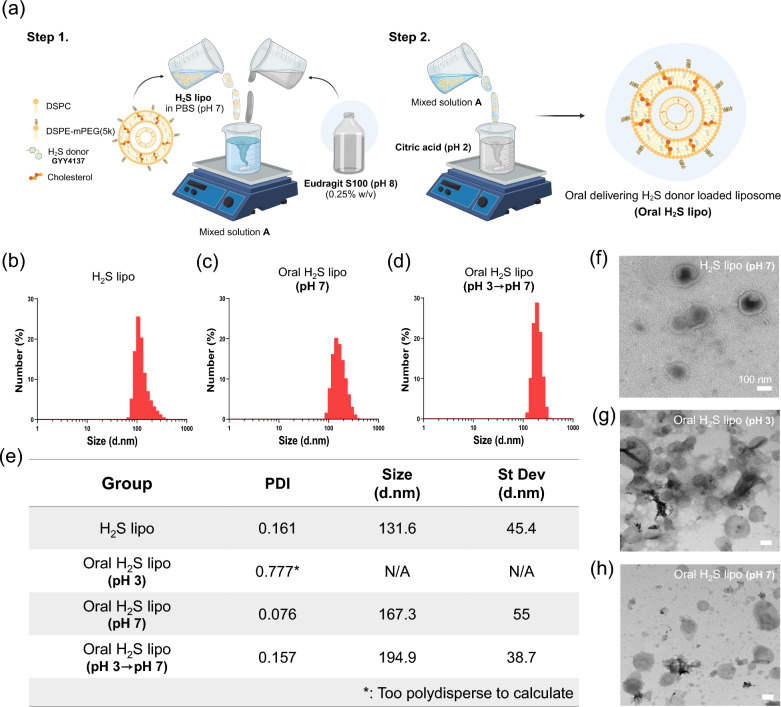
Characteristics of H_2_S lipo and Oral H_2_S lipo. (**a**) Schematic of pH-driven method for synthesizing Oral H_2_S lipo. Hydrodynamic size of H_2_S lipo (**b**) and pH-dependent Oral H_2_S lipo (**c, d**) analyzed by DLS. TEM images of H_2_S lipo (**e**) and pH-dependent Oral H_2_S lipo. (**f, g**)

A schematic illustration of the pH-driven method used to synthesize Oral H₂S lipo is presented in Fig. 1a. To prepare the Eudragit solution, a pH 8 buffer was prepared by mixing 0.2 M sodium phosphate dibasic and 0.1 M citric acid. An appropriate amount of Eudragit S100 powder was dissolved in the pH 8 buffer to achieve final concentrations of 0.01%, 0.05%, 0.1%, and 0.25% (w/v). Each Eudragit solution was subsequently mixed with the previously prepared H₂S lipo at a 1:1 volume ratio to coat its surface. During this process, H₂S lipo was gradually added dropwise into the continuously stirred Eudragit solution to ensure thorough mixing. Stirring was continued for an additional 30 min after the dropwise addition. Subsequently, the resulting mixture of H₂S lipo and Eudragit was added dropwise into continuously stirred citric acid buffer (pH 2) at a 1:1 volume ratio, and stirring was continued for 30 min to stabilize the coating under acidic conditions. Finally, unbound Eudragit was removed by syringe filtration, yielding the final product: Oral H₂S lipo. The GYY4137 (an H₂S donor) release test from Oral H₂S lipo under different pH conditions was performed by mixing the purified formulation with pH buffer solutions. As with the coating step, the formulation was gradually added dropwise into continuously stirred buffer solutions (Citric acid [pH 2] or PBS [pH 7]) at a 1:1 volume ratio, followed by an additional 30 min of stirring. In all in vitro and in vivo experiments, except for the GYY4137 release study, Oral H₂S lipo was prepared using 0.25% Eudragit. For fluorescence imaging, both H₂S lipo and Oral H₂S lipo were labeled with DiR by incorporating 5 µM DiR into the lipid mixture prior to solvent removal, and the subsequent steps were carried out as described above.

### Validation of the pH-driven method for Oral H₂S lipo characterization

The Oral H₂S lipo synthesized via the pH-driven method was validated by exposing it to different pH conditions and transitions, followed by analysis using DLS and cryo-TEM. Particle size and polydispersity index (PDI) were evaluated under pH 3 and pH 7 using DLS. The formulation was mixed with buffer solutions at pH 3 and pH 7 in a 1:9 (v/v) ratio and incubated for approximately 1 h prior to analysis. For the pH transition experiment (pH 3 to pH 7), the formulation was first incubated at pH 3 for 1 h, then transferred to pH 7 and further incubated for 1 h prior to sampling. To complement the DLS analysis, TEM was used to visualize morphological changes induced by the pH transition, using a single formulation batch. Following incubation at pH 3, a portion was sampled for TEM imaging, while the remaining material was then transferred to pH 7, incubated under the same conditions, and sampled again for analysis. Imaging at both time points enabled direct comparison of pH-induced morphological changes within the same sample.

### In vitro stability test of Oral H₂S lipo under different pH conditions

The pH-dependent stability of Oral H₂S lipo was evaluated by mixing the formulations with buffer solutions at pH 2, 4, and 7 in a 1:9 (v/v) ratio. Samples were incubated at room temperature for up to 14 days under each condition to simulate gastrointestinal environments. At each measurement point (days 0, 3, 7, 10, and 14), a portion of the sample was collected and adjusted to pH 7 in order to dissolve the Eudragit S100 coating, thereby releasing the encapsulated Oral H₂S lipo for subsequent particle size analysis. The samples were then allowed to stand at room temperature for 1 h prior to particle size measurement. The percentage change in size over the incubation period was calculated using the following equation:

$$\begin{aligned}&\text{Size change }({\%})\\&=\left(\frac{\text{Final size}\left(\text{Day }14\right)-\text{Initial size}(\text{Day }0)}{\text{Initial size}(\text{Day }0)}\right)\times 100\end{aligned}$$

### Loading efficiency and acidic stability of Oral H₂S lipo

The loading efficiency of Oral H₂S lipo was determined by detecting H₂S released from disrupted liposomes via precipitation with silver nitrate (AgNO₃). To disrupt the liposomal structure, Oral H₂S lipo was first lyophilized, and the freeze-dried sample was treated with methanol (MeOH) and dimethyl sulfoxide (DMSO) at a 1:2 (v/v) ratio. The mixture was subsequently centrifuged at 12,000 rpm for 60 min to separate the components. Following centrifugation, the pelleted lipids and the supernatant containing GYY4137 (an H₂S donor) were collected. The supernatant was reacted with AgNO₃, resulting in the formation of an insoluble silver sulfide (Ag₂S) precipitate. The amount of Ag₂S was quantified colorimetrically to determine GYY4137 loading efficiency. For the assay, the supernatant, AgNO₃, and methanol were mixed in a 1:1:3 (v/v/v) ratio and incubated at room temperature for 1 h to allow complete H₂S release. A calibration curve for GYY4137 was established **(Fig. S2)**, and samples were analyzed for absorbance at 405 nm (optical density, O.D. of Ag₂S). Loading efficiency (%) was calculated using the following equation:

$$\begin{aligned}&{H}_{2}\text{S loading efficiency}\,({\%}) \\&=\left(\frac{Mass\, of\, GYY4137\, in\, the\, sample\, (converted\, from\, measured\, O.D.)}{Initial\, mass\, of\, GYY4137}\right)\\&\quad\times 100\end{aligned}$$

To evaluate acidic stability, samples were mixed with pH 2 buffer (1:9, v/v) and incubated at room temperature. GYY4137 release was monitored over 8 days (0, 2, 4, 6, 8 days). At each time point, aliquots were mixed with AgNO₃ (1:1, v/v), incubated for 1 h, and analyzed at 405 nm. A GYY4137 solution was analyzed under the same conditions for comparison. The percentage stability change was calculated as:

$$\begin{aligned}&\text{Loading stability }({\%}\, \text{change}) \\&=\left(100\%(Initial\, mass\, of\, GYY4137)\right)\\&\quad-\left\{\left(\frac{Initial\, mass\, of\, GYY4137-Mass\, of\, GYY4137\, in\, the\, sample\, (converted\, from\, measured\, O.D.)}{Initial\, mass\, of\, GYY4137}\right) \times 100\right\}\end{aligned}$$

### Evaluation of in vitro cell viability

HFF and RAW 264.7 cells were seeded in 96-well plates at 1 × 10⁶ cells/mL and incubated for 24 h. Cells were cultured in DMEM containing 10% (v/v) FBS and 1% (v/v) antibiotics in a humidified incubator at 37 °C with 5% CO₂. GYY4137 (an H₂S donor), H₂S lipo, and Oral H₂S lipo were serially diluted to equivalent GYY4137 concentrations (0–120 µM). Bare oral lipo (without GYY4137) and bare lipo (without both GYY4137 and Eudragit S100) were prepared and diluted to match lipid concentrations (0–5.18 mM).

The diluted samples were added to pre-incubated cells and incubated for 6 h. After incubation, 100 µL of MTT solution (0.5 mg/mL in PBS) was added and cells were incubated for 4 h. Formazan crystals were dissolved with 100 µL of DMSO, and cell viability was determined by measuring absorbance at 540 nm using a microplate reader.

### pH-dependent GYY4137 release test

The time-dependent H₂S release behavior of GYY4137 from Oral H₂S lipo under various Eudragit concentrations and pH conditions was evaluated by time-course mass spectrometry. Figure 4a illustrates the setup for H₂S release measurement at 0, 30, 60, and 90 min. The percentage of H_2_S release from GYY4137 was calculated as the ratio of the intensity of hydrolyzed GYY4137 (m/z 187.016) to the total intensity of hydrolyzed (m/z 187.016) and intact GYY4137 (m/z 288.028), enabling precise assessment of their time-dependent stability and release behavior across the two pH conditions. Experimental groups included Oral H₂S lipo coated with Eudragit at 0.01%, 0.025%, 0.05%, and 0.1% (w/v), with free GYY4137 and H₂S lipo serving as controls. All samples were evaluated under acidic (pH 2) and neutral (pH 7) environments to compare their release profiles. Mass spectrometric analyses were conducted using a Q Exactive Orbitrap mass spectrometer (Thermo Fisher Scientific, USA) in negative ion mode at a resolution of 140,000 (m/z 100–500). Instrument settings included a capillary temperature of 300 °C, capillary voltage of –44 V, and spray voltage of 5 kV applied to the ESI probe.

### Ex-vivo fluorescence imaging in DSS-induced colitis mice

A colitis mouse model was established by administering 2.5% (w/v) dextran sulfate sodium (DSS) in 3 × DW as drinking water to eight-week-old C57BL/6 mice for six consecutive days, followed by regular water for four days. On day 10, one day after DSS treatment, mice were orally gavaged with DiR-Oral H₂S lipo (experimental group) or saline, DiR, or DiR-H₂S lipo (control groups). Mice were euthanized 12 h later, and the heart, lungs, liver, spleen, colon, and small intestine were collected. Ex vivo organ imaging was performed using an in vivo imaging system (IVIS), and fluorescence intensity was quantified with Living Image 4.7.4 software.

### Therapeutic assessment in DSS-mediated colitis model

Female C57BL/6 mice aged 8 weeks were used in all experimental groups. Mice in the normal group received regular drinking water, while colitis was induced in the saline and Oral H₂S lipo groups by administering 2.5% DSS solution as drinking water. DSS was provided for 6 consecutive days, followed by 4 days of regular water. Saline, GYY4137 (200 μM),and Oral H₂S lipo (200 μM) were administered once daily via oral gavage **(**Fig. 6a**)**. Dosing concentration of GYY4137 and Oral H_2_S were determined from references [30, 54]. Body weight was monitored daily to assess disease progression, and percentage weight change was calculated using the following formula:

$$\begin{aligned}&\text{Weight change }({\%}) \\&=\left(\frac{\text{Body weight at each time point}}{\text{Initial body weight}}\right)\times 100\end{aligned}$$

At the end of the experiment, all colitis model mice were euthanized, and the entire colon was extracted. The colons were straightened from the cecum and measured using a ruler to assess inflammation-induced shortening. For colon weight and histological analyses, the distal 3 cm of the colon was excised. Colon weight was used to calculate the weight-to-length ratio, and H&E staining was performed using the same distal segment to evaluate tissue damage. Inflammation scoring was conducted based on the methods described in reference [55], with a total score ranging from 0 to 11. The scoring system included four categories: severity of inflammation (1–4), extent of inflammation (1–3), epithelial hyperplasia (0 or 1), and ulceration (0 or 3).

### Immunofluorescence

**Scheme 1:**
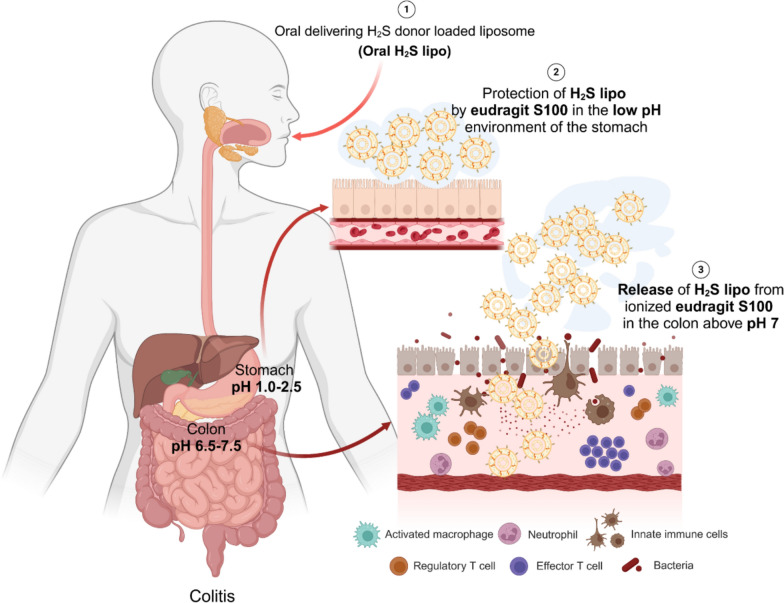
Mechanism of action of Oral H_2_S liposomes in the treatment of IBD

The colonic tissues were fixed in 4% paraformaldehyde (PFA), embedded in paraffin, and sectioned at a thickness of 4 μm into glass slides. Deparaffinization and rehydration were carried out by sequential incubation in xylene, followed by different ethanol concentration. Antigen retrieval was achieved by heating the sections in 10 mM sodium citrate buffer (pH 6.0) at 100 °C for 20 min. After permeabilization with 0.5% Triton X-100, the samples were incubated with primary antibodies against FOXP3 (Invitrogen, lot 2,349,824), TNF-α (Invitrogen, lot ZK4531382A), and IL-1β (Invitrogen, lot ZJ4505521). For visualization of TNF-α and IL-1β, tyramide signal amplification (TSA) was applied using Alexa Fluor™ 555 Tyramide (Invitrogen, lot 2,881,766). Fluorescence images were obtained with a confocal microscope, and signal intensities were quantified using ImageJ software. Scheme 1.

## Results

### Characteristics of Oral H_2_S lipo

To investigate the physicochemical characteristics of the developed oral hydrogen sulfide donor-loaded liposomes (Oral H₂S lipo), we analyzed their hydrodynamic diameter and polydispersity index (PDI) under simulated gastrointestinal pH conditions (pH 3 and pH 7). H₂S lipo without Eudragit S100 coating exhibited a hydrodynamic diameter of 131.6 ± 45.4 nm and a PDI of 0.161, consistent with previously reported data **(**Fig. 1b, e**)** [54]. In contrast, Oral H₂S lipo showed no measurable particle size at pH 3, and its PDI was 0.777 **(**Fig. 1e**)**. These results suggest that the high polydispersity under acidic conditions arises from Eudragit S100-mediated aggregation. At pH 7, Oral H₂S lipo exhibited a particle size of 167.3 nm and a PDI of 0.076, comparable to H₂S lipo **(**Fig. 1c, e**)**. This indicates that Eudragit S100 dissolves at pH 7, enabling accurate characterization of particle size and dispersity. To further confirm this behavior, a pH transition experiment (pH 3 → pH 7) was conducted. The formulation was incubated at pH 3 and then transferred to pH 7 before analysis. Upon transition, the formulation exhibited a particle size of 194.9 nm and a PDI of 0.157 (Fig. 1d, e), indicating partial recovery of its original characteristics. These findings demonstrate that the Eudragit S100 coating undergoes pH-dependent structural transitions—condensing into a protective layer under acidic conditions and dissolving under neutral conditions—enabling reversible disassembly and re-dispersion of the liposomes. The morphological characteristics of the formulations were further examined by cryogenic transmission electron microscopy (cryo-TEM). For H₂S lipo, the observed particle size and morphology closely matched the dynamic light scattering (DLS) results, confirming consistency between the two methods (Fig. 1f). To assess pH-dependent structural transitions, Oral H₂S lipo from a single batch was incubated at pH 3, with a portion imaged directly and the remainder transferred to pH 7. At pH 3, the formulation exhibited distinct polymeric aggregation consistent with Eudragit S100 condensation (Fig. 1g), whereas following pH neutralization, the liposomes regained a uniform morphology similar to H₂S lipo (Fig. 1h). These observations visually corroborate the DLS findings and validate the pH-responsive behavior of the system. Collectively, Oral H₂S lipo undergoes reversible, pH-triggered structural transitions: condensing into a protective aggregated state under acidic conditions and redistributing into discrete liposomes under neutral conditions. This behavior highlights the functional role of the Eudragit S100 coating in protecting the formulation during gastric transit while enabling targeted release in the colon, which is essential for effective oral delivery of H₂S donors in IBD therapy.

### Comprehensive evaluation of Oral H₂S lipo: stability, loading efficiency, and retention under simulated gastrointestinal conditions

**Fig. 2:**
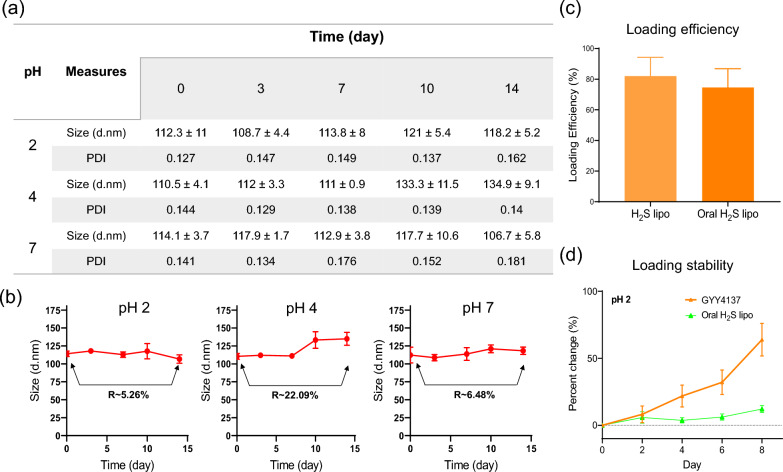
Liposome stability test and loading efficiency. (**a**) Stability test results of Oral H_2_S lipo at pH 2,4, and 7. (**b**) Percentage change in size between day 0 and day 14. (**c**) Comparison of GYY4137 loading efficiency between H_2_S lipo and Oral H_2_S lipo. (**d**) Time-dependent comparison of loading stability between free GYY4137 and Oral H₂S lipo at pH 2

The stability of Oral H₂S lipo was evaluated over 14 days under simulated gastrointestinal conditions (pH 2, 4, and 7). Particle size and polydispersity index (PDI) were monitored by dynamic light scattering (DLS), and particle size distribution (PSD) histograms were analyzed at each time point **(**Fig. 2a, b;Fig. S1). According to FDA guidelines, nanoparticle systems generally require a PDI below 0.3; exceeding this threshold indicates a broad size distribution and potential instability. In practice, a PDI of 0.05–0.2 is considered optimal [56, 57]. Oral H₂S lipo maintained PDI values between 0.127 and 0.181 across all pH conditions throughout the 14-day period, indicating consistent uniformity and formulation stability. Particle size changes were monitored under simulated gastrointestinal conditions. To mimic pH transitions in the gastrointestinal tract, samples were incubated at pH condition pH 2, 4, or 7 for up to 14 days. At each time point, aliquots were adjusted to pH 7 and allowed to stand before particle size measurement. As a result, minimal variation was observed over 14 days, with changes between day 0 and day 14 of 5.26% (pH 2), 22.09% (pH 4), and 6.48% (pH 7) **(**Fig. 2b**)**. Taken together, the analysis of PDI and particle size over 14 days revealed that Oral H₂S lipo maintained stability not only under neutral conditions (pH 7) but also under harsher environments, including strongly acidic (pH 2) and mildly acidic (pH 4) conditions. These findings suggest that the Eudragit S100 coating effectively protects Oral H₂S lipo by condensing under acidic conditions. GYY4137 (an H₂S donor) loading efficiency of Oral H₂S lipo was determined by a precipitation reaction with AgNO₃. H₂S lipo achieved 82.02%, while the Oral H₂S lipo reached 74.65%, indicating comparable loading despite the Eudragit S100 coating, Oral H₂S lipo retained a high GYY4137 loading efficiency comparable to that of H₂S lipo **(**Fig. 2c**)**. To evaluate retention under acidic conditions, the release of H_2_S from Oral H₂S lipo was monitored at pH 2 over 8 days. The formulation exhibited only 12.13% cumulative release of H_2_S, representing a ~ fivefold reduction compared to free GYY4137, which showed 60% release over the same period **(**Fig. 2d**)**. This pronounced reduction in premature release of H_2_S underscores the protective role of the Eudragit coating in minimizing drug leakage under gastric conditions.

Collectively, these findings demonstrate that Oral H₂S lipo combines excellent colloidal stability, high drug-loading efficiency, and acid resistance—key attributes of a material-driven platform designed for colon-targeted oral drug delivery.

### In vitro evaluation of cytotoxicity and biocompatibility of Oral H₂S lipo

**Fig. 3:**
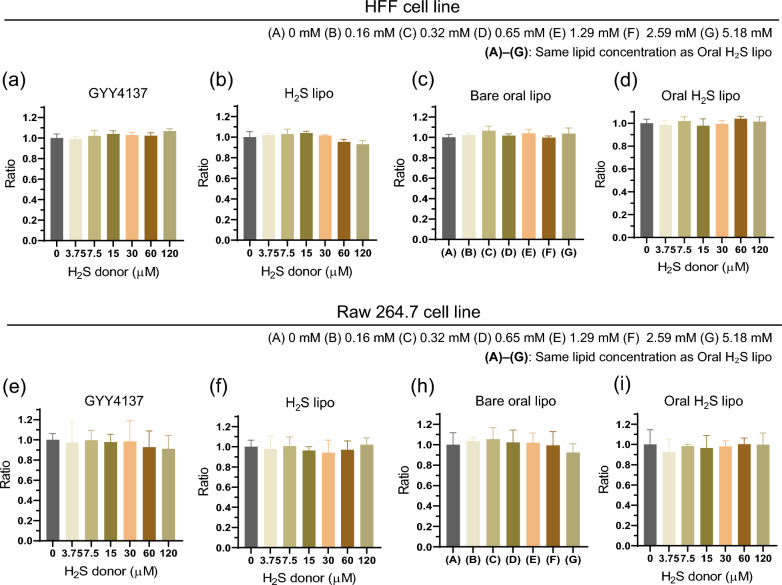
Cell viability test via MTT assay in HFF and raw 264.7 cell lines. (**a**) Cell viability test in HFF cell line treated with GYY4137, H_2_S lipo, and Oral H_2_S lipo (GYY4137 concentration: 0–120 µM) and bare oral lipo (lipid concentration: 0–5.18 mM). (**b**) Cell viability test in raw 264.7 cell line treated GYY4137, H_2_S lipo, and Oral H_2_S lipo (GYY4137 concentration: 0–120 µM) and bare oral lipo (lipid concentration: 0–5.18 mM)

The in vitro safety of Oral H_2_S lipo was assessed using an MTT (3-(4,5-dimethylthiazol-2-yl)−2,5-diphenyltetrazolium bromide) assay in HFF and RAW 264.7 cell lines. For comparison, GYY4137 (an H₂S donor), H₂S lipo, bare lipo, and bare oral lipo were also evaluated. Cells were treated with GYY4137, H₂S lipo, and Oral H₂S lipo at equivalent GYY4137 concentrations (0–120 µM), while bare lipo and bare oral lipo were tested at lipid concentrations matching those of Oral H₂S lipo (0–5.18 mM). Across the tested range, GYY4137, H₂S lipo, and Oral H₂S lipo exhibited no detectable cytotoxicity in either cell line **(**Fig. 3a, b, d, e, f, i**)**. Similarly, bare lipo (without GYY4137 and Eudragit S100) and bare oral lipo (without GYY4137) were non-toxic at a lipid concentration equivalent to those of Oral H₂S lipo (Fig. S3a, b; Fig. 3c, h**)**. These results demonstrate the high biocompatibility of Oral H₂S lipo, supporting its potential as a safe platform for colon-targeted oral drug delivery.

### pH-dependent release profile of GYY4137

**Fig. 4:**
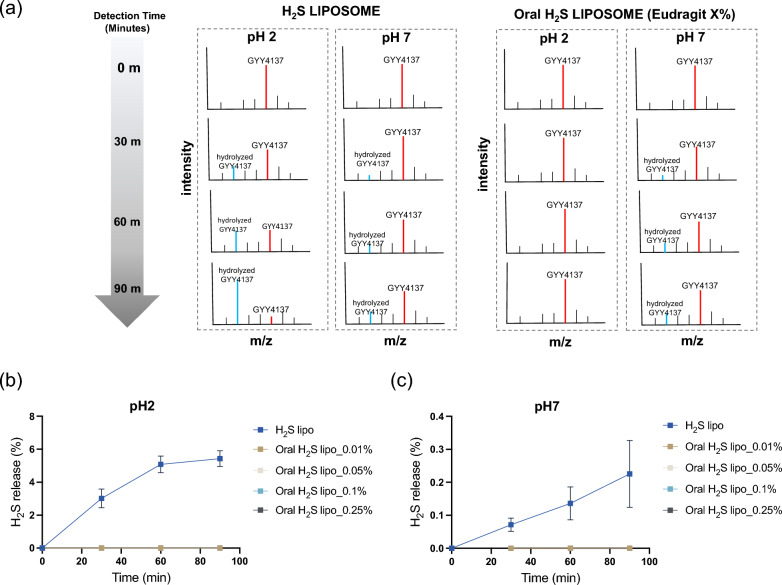
pH-dependent GYY4137 release test. (**a**) Scheme of the GYY4137 release test procedure (**b**) H_2_S release test at pH 2 and pH 7 for each group: GYY4137, H_2_S lipo, and Oral H_2_S lipo with Eudragit S100 concentrations of 0.01%, 0.05%, 0.1%, and 0.25%. H_2_S release (%): ratio of the intensity of hydrolyzed GYY4137 (*m/z* 187.016) to the total intensity of hydrolyzed (*m/z* 187.016) and intact GYY4137 (*m/z* 288.028)

To quantitatively characterize the pH-responsive release profile of Oral H₂S zlipo, we performed mass spectrometric analysis of GYY4137 hydrolysis under simulated gastric (pH 2) and colonic (pH 7) conditions. Samples were prepared with varying concentrations of Eudragit S100 (0.01%, 0.05%, 0.1%, and 0.25% w/v) to evaluate the coating’s protective efficacy (Fig. 4a**)**. H_2_S release was quantified by calculating the ratio of the intensity of hydrolyzed GYY4137 (*m/z* 187.016) to the total intensity of both hydrolyzed (*m/z* 187.016) and intact GYY4137 (*m/z* 288.028). The relative difference in the ionization efficiency between the intact and hydrolyzed forms of GYY4137 was taken into account in these analyses. In H₂S lipo, the proportion of hydrolyzed GYY4137—corresponding to released H₂S—increased over time under both pH conditions, with a markedly higher release at pH 2 compared to pH 7 (5.41 ± 0.48% vs. 0.23 ± 0.10%, p < 0.001) (Fig. 4b, c; Fig. S4). These results indicate that liposomal encapsulation can slow GYY4137 hydrolysis but is insufficient to prevent premature release under acidic conditions. In contrast, all Oral H₂S lipo formulations containing Eudragit S100 completely suppressed GYY4137 release at pH 2, with no detectable intact or hydrolyzed GYY4137, indicating strong protective capability of the coating. Upon exposure to neutral pH (pH 7.0), Oral H₂S lipo exhibited a gradual, time-dependent release profile similar to H₂S lipo **(**Fig. 4c**)**. This pH-sensitive behavior was consistent across the entire range of tested Eudragit concentrations (0.01%–0.25%), confirming the robustness and tunability of the coating. The use of intensity ratio analysis in high-resolution mass spectrometry enabled precise, time-resolved quantification of GYY4137 hydrolysis, validating the reproducibility and sensitivity of the platform. These findings not only confirm the critical role of Eudragit S100 in stabilizing the formulation under gastric conditions and facilitating release in the colon, but also indicate that effective protection can be achieved with minimal polymer content. Although 0.25% Eudragit was employed in our other experiments [58], our data indicate that comparable performance is attainable with as little as 0.01%, a formulation advantage that may improve safety, reduce material requirements, and simplify future clinical translation.

### Colon-targeted biodistribution of DiR-Oral H₂S lipo in a DSS-induced colitis model

**Fig. 5:**
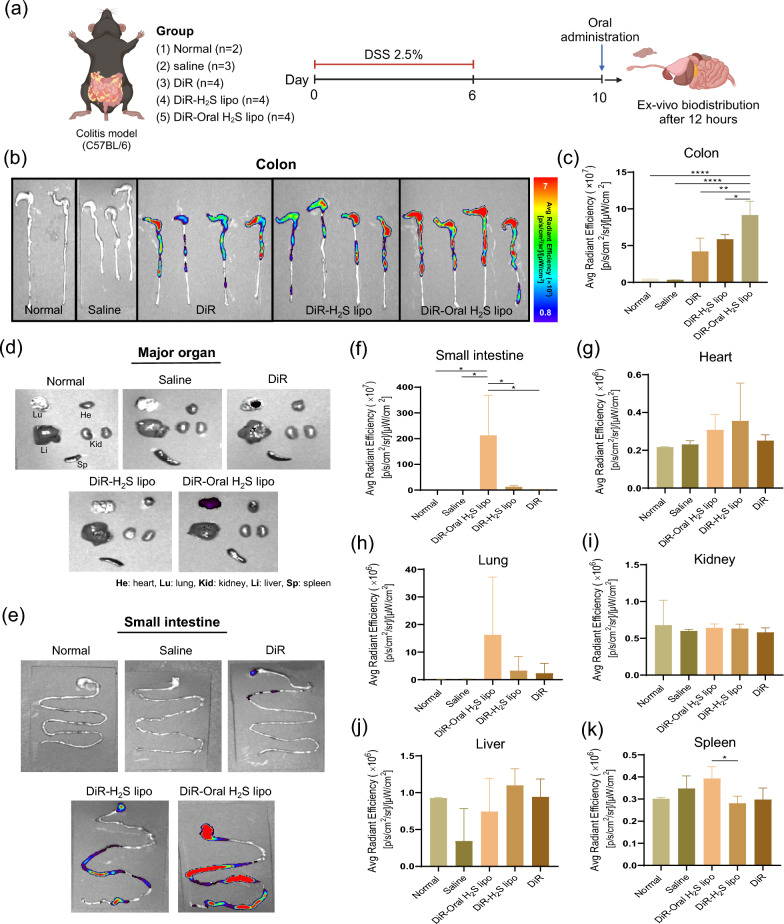
Ex-vivo imaging results at 12 h after oral administration in colitis model for treatment and control groups using the in vivo imaging system (IVIS). (**a**) Schematic of the in vivo experimental procedure. (**b**) Ex-vivo fluorescence Imaging of colons. (**c**) Fluorescence quantification in the colon via IVIS imaging. (**d**) Ex-vivo fluorescence imaging of major organs and (**e**) small intestines. Fluorescence quantification analysis in small intestines (**f**) and major organs: Heart (**g**), Lung (**h**), Kidney (**i**), Liver (**j**), and spleen (**k**) via IVIS imaging. *: P < 0.05, **: P < 0.01, ***: P < 0.001, ****: P < *0.0001*

Biodistribution of DiR-Oral H₂S lipo was compared with control groups (normal, saline, DiR, and DiR-H₂S lipo) using ex vivo imaging in a DSS-induced colitis mouse model **(**Fig. 5a**)**. During formulation, Eudragit S100—a pH-sensitive polymer widely applied in colon-targeted delivery due to its solubility at pH ≥ 7 [59, 60], was incorporated. We therefore hypothesized that DiR-Oral H₂S lipo would remain intact in the acidic environment of the stomach but dissolve in the colon (pH ≥ 7), thereby improving colon targeting. Ex vivo imaging was first conducted 12 h after administration. As expected, the experimental formulation exhibited the highest level of accumulation in the colon of DSS-induced colitis mice compared to all control groups **(**Fig. 5b**)**. Image analysis revealed that the intensity of DiR-Oral H₂S lipo was significantly higher than that of the normal and saline groups (P < 0.0001), as well as DiR (P < 0.01) and DiR-H₂S lipo (P < 0.05) **(**Fig. 5c**)**. Fluorescence signals were also observed in the cecum, likely reflecting its anatomical proximity to the colon and retrograde movements that prolong retention [61–64]. Accordingly, DiR-Oral H₂S lipo group showed the strongest cecal signal among groups **(**Fig. 5b**; **Fig. S5e). In the small intestine, DiR-Oral H₂S lipo exhibited significantly higher fluorescence than all controls (P < 0.05) **(**Fig. 5e, f**)**, likely due to the protective effect of the Eudragit S100 coating, which prevented premature degradation and enabled transit to the colon. Importantly, strong colon-specific accumulation confirmed that the formulation remained stable through the small intestine and was effectively delivered to the colon. Analysis of major organs (heart, kidneys, and liver) showed no detectable signals in any group **(**Fig. 5d, g, i, j**)**. In the lungs, DiR-Oral H₂S lipo showed slightly higher intensity than DiR-H₂S lipo, though not statistically significant **(**Fig. 5d, h**)**. In contrast, fluorescence intensity in the spleen was significantly higher for DiR-Oral H₂S lipo than for DiR-H₂S lipo (P < 0.05) **(**Fig. 5d, k**)**. The time required for orally administered drugs to reach the colon typically ranges from approximately 6 to 70 h, with notably shorter transit times reported in colonic diseases [65–67]. Based on this, additional in vivo and ex vivo imaging was conducted 6 h post-administration to assess whether DiR-Oral H₂S lipo effectively targets the colon at this early time point. In vivo imaging revealed stronger fluorescence signals in the colonic region compared with control groups (Fig. S5a). Similarly, ex vivo imaging demonstrated higher colonic accumulation of DiR-Oral H₂S lipo relative to controls, with strong signals detected in the cecum (Fig. S5b, d). Quantitative analysis showed statistical significance versus the normal group, although no significant difference were observed relative to DiR or DiR-H₂S lipo (Fig. S5c). Notably, the ex vivo data at 6 h exhibited a trend consistent with the 12-h results, suggesting that DiR-Oral H₂S lipo begins to accumulate in the colon as early as 6 h post-administration and demonstrates colon-targeting ability even at this early time point. Collectively, these findings demonstrate that Oral H₂S lipo exhibits rapid and preferential accumulation in the inflamed colon as early as 6 h after administration, with minimal off-target distribution. This biodistribution profile validates the role of the Eudragit S100 coating as a pH-responsive trigger and highlights the potential of the platform as a precise, non-invasive strategy for colon-specific delivery of H₂S donors in IBD therapy.

### Evaluation of therapeutic effects in a DSS-induced colitis mice model

**Fig. 6:**
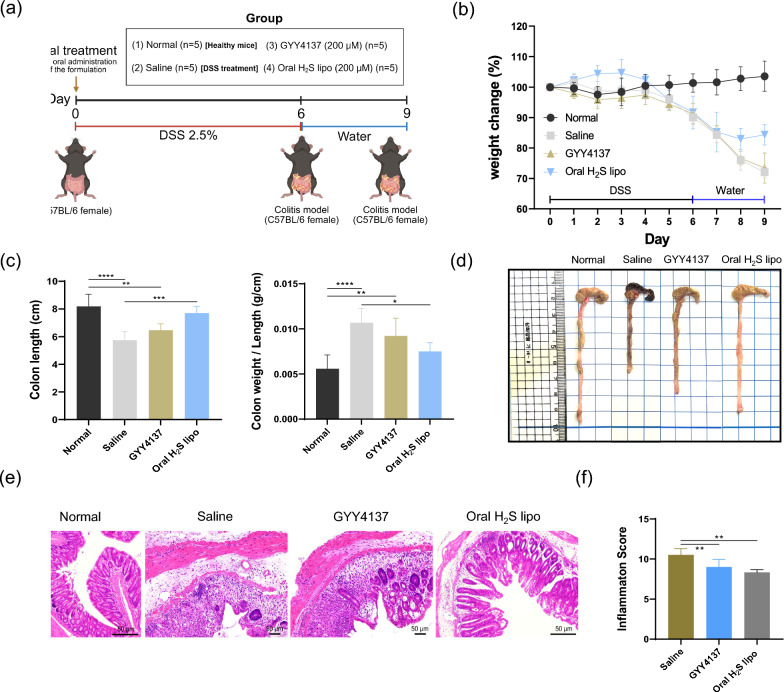
Therapeutic efficacy of Oral H₂S lipo in a DSS-induced colitis model. (**a**) Schematic of the treatment protocol. Colitis was induced in C57BL/6 mice using 2.5% DSS solution. All groups received daily oral gavage treatments, including both treatment and control groups. (**b**) Effect of Oral H₂S lipo on body weight changes in DSS-induced colitis mice (n = 6). Weight change (%) was calculated relative to day 0. (**c**) Comparison of colon length and colon weight-to-length ratio (g/cm) (n = 6). (**d**) Representative images of colons from each group. (**e**) Histological H&E staining of colonic tissues from each group after treatment. (**f**) Effect on inflammation scores (n = 6). *: P < 0.05, **: P < 0.01, ***: P < 0.001. ****: P < *0.0001*

**Fig. 7:**
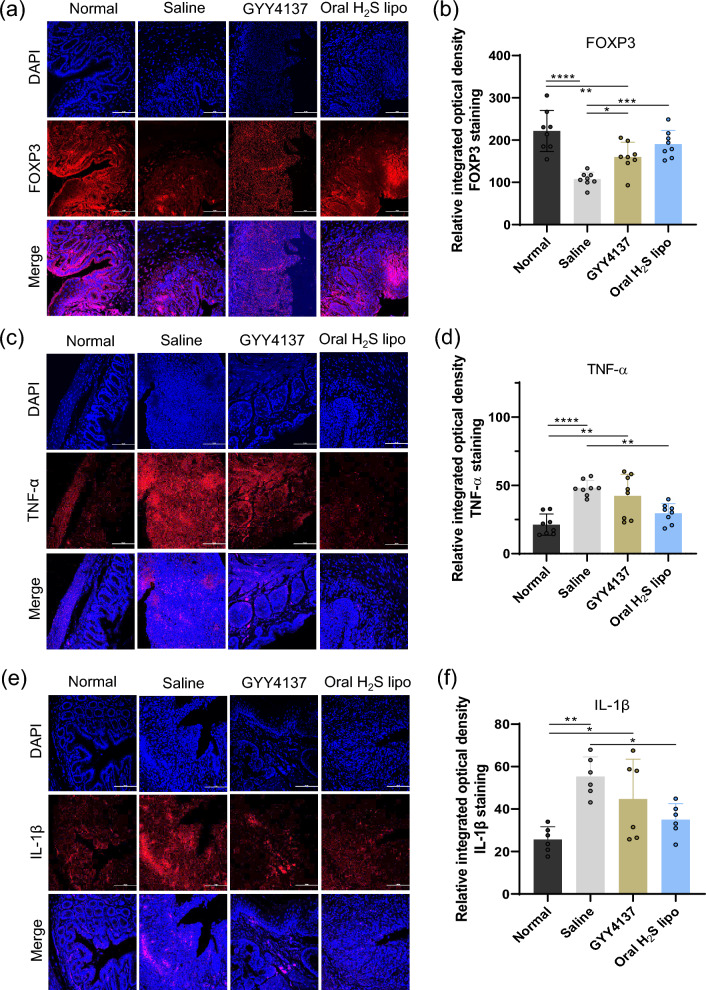
Immunofluorescence analysis of FOXP3 and pro-inflammatory cytokines (TNF-α and IL-1β) in colonic tissues. Confocal images of (**a**) FOXP3, (**c**) TNF-α, and (**e**) IL-1β expression in each group (DAPI: nuclear staining; red fluorescence from Cy5.5-labeled antibodies indicates the target proteins). Corresponding quantitative fluorescence intensity analyses are presented in (**b**), (**d**), and (**f**), respectively (n = 8 for FOXP3 and TNF-α; n = 6 for IL-1β).*: P < 0.05, **: P < 0.01, ***: P < 0.001. ****: P < 0.0001

To assess the therapeutic potential of Oral H₂S lipo, a DSS-induced colitis mouse model was established, and animals were randomly assigned to three groups: normal, saline-treated, and Oral H₂S lipo-treated (200 μM) (Fig. 6a). Oral H₂S lipo was administered once daily via oral gavage for ten consecutive days. During the treatment period, body weight change (%) was monitored as a clinical indicator of colitis severity. On day 10, mice treated with Oral H₂S lipo showed less body weight loss (79.90 ± 14.55%) compared to the saline group (72.13 ± 12.63%), although the difference was not statistically significant (One-way ANOVA with Tukey’s post hoc test, p = 0.686) (Fig. 6b). Macroscopic evaluation of colon length revealed a longer colon in the Oral H₂S lipo group relative to the saline group (6.47 ± 0.50 cm vs. 5.53 ± 0.55 cm), suggesting partial protection against inflammation-induced tissue contraction, although not statistically significant (p = 0.152) (Fig. 6c, d). Histological examination of H&E-stained colon sections further supported the therapeutic effect of Oral H₂S lipo. The saline group exhibited marked epithelial disruption and extensive immune cell infiltration, whereas the Oral H₂S lipo showed preserved epithelial architecture with reduced inflammatory cell presence, more closely resembling normal colonic morphology (Fig. 6e). Notably, the histological inflammation score was significantly lower in the Oral H₂S lipo group compared to the saline group (p < 0.05) (Fig. 6f**),** indicating attenuation of mucosal inflammation. And, in blood biochemical analysis for assessing toxicity of Oral H_2_S lipo, serum alanine aminotransferase (ALT), aspartate aminotransferase (AST), blood urea nitrogen (BUN), and creatinine (CREA) measured on day 10 did not differ significantly from the Normal group, indicating no apparent toxicity associated with the Oral H_2_S lipo (Fig. S6). Also, in histological images of H&E-stained sections from major organs (liver, spleen, kidney, lung, heart, and stomach), no remarkable abnormalities were found in the examined organs (Fig. S7). And we evaluated the immunomodulatory effects of Oral H_2_S lipo at the histological level. Specifically, we assessed the expression of the regulatory T cells (T_regs_) (Fig. 7a) marker FOXP3 and the pro-inflammatory cytokine markers TNF-α (Fig. 7b) and IL-1β (Fig. 7c) in fixed colonic tissues. The results showed that FOXP3 expression was significantly increased in both the GYY4137- and Oral H_2_S lipo–treated groups compared to the saline-treated group (P < 0.05 and P < 0.001, respectively). However, a significant reduction in pro-inflammatory cytokine expression was observed only in the Oral H_2_S lipo–treated group (TNF-α: P < 0.01; IL-1β: P < 0.05). Collectively, these findings indicate that Oral H_2_S lipo, upon colonic accumulation in the DSS-induced colitis model, successfully inactivated the inflammasome, alleviated innate immune responses, and promoted T_regs_ cell differentiation. In sum up, these findings suggest that Oral H_2_S lipo mitigates colonic inflammation and preserves tissue integrity in DSS-induced colitis, supporting its potential as an effective oral nanomedicine for IBD.

## Discussion

Recently, significant progress has been made in understanding hydrogen sulfide (H₂S) as an endogenous gasotransmitter. Similar to nitric oxide (NO) and carbon monoxide (CO), H₂S is now recognized as a key signaling molecule that regulates various physiological and pathophysiological processes [68–70]. Although it was initially regarded as a toxic gas due to its harmful effects at high concentrations, its role has been redefined following the discovery of its neuromodulatory functions in 1996, and it has since been recognized for its diverse roles, particularly in maintaining homeostasis and suppressing inflammation within the nervous, gastrointestinal, cardiovascular, renal, and hepatic systems [71–74].

Specifically, in the gastrointestinal tract, H₂S plays several critical roles. First, it contributes to the maintenance of intestinal homeostasis [75, 76]. Second, it helps preserve the integrity of colonic epithelial cells and the mucus barrier [77, 78]. Third, H₂S exhibits anti-inflammatory properties by suppressing excessive immune responses [79, 80]. Moreover, H₂S participates in the regulation of bacterial biofilm formation in the gut, thereby potentially alleviating chronic inflammation. Mechanistically, it may exert its effects either directly on the gut microbiota or indirectly by influencing biofilm structural stability [25, 81]. For instance, physiologically relevant concentrations of H₂S promote mucus secretion and help maintain the expression of epithelial tight junction proteins, such as ZO‑1, claudin, and occludin. These effects help prevent the direct penetration of microbes and harmful toxins into the colonic epithelium, thereby contributing to gut barrier and biofilm stability [30, 82].

However, these physiological functions of H₂S are maintained only when its concentration is tightly regulated. Conversely, dysregulated H₂S levels may contribute to pathophysiological responses [25]. At low concentrations, the regenerative capacity of the mucus layer and epithelial cells is reduced, and its anti-inflammatory effects are diminished, potentially leading to intestinal inflammation [83]. In contrast, excessive H₂S accumulates in the mitochondria of colonic epithelial cells (colonocytes), where it inhibits cytochrome c oxidase, disrupts ATP production, and impairs cellular energy metabolism [84]. This leads to impaired epithelial function and increased vulnerability of the mucus barrier, promoting the invasion of pathogenic microorganisms and exacerbating intestinal inflammation [85, 86].

This dual nature of H₂S is largely attributed to imbalances in its concentration, which are thought to be influenced by three key factors: (1) the limited capacity of intracellular H₂S detoxification and diffusion mechanisms, (2) alterations in the gut microbiota, and (3) dysregulation of endogenous H₂S metabolism [87]. First, the diffusion and detoxification mechanisms of H₂S play a critical role in regulating its intracellular concentration. Colonocytes employ mitochondrial H₂S-oxidizing enzymes, such as sulfide quinone oxidoreductase (SQOR), to detoxify H₂S and incorporate it into energy metabolism [88]. However, chronic exposure to high concentrations of H₂S may overwhelm these detoxification pathways, resulting in its intracellular accumulation in colonocytes. This accumulation inhibits mitochondrial cytochrome c oxidase, thereby disrupting ATP production. Consequently, ATP depletion impairs cellular function and reduces the expression of tight junction proteins, which compromises the integrity of the intestinal mucosa [76, 89, 90]. This facilitates the translocation of pathogens and endotoxins, thereby aggravating inflammation and reinforcing the pathological cycle [91, 92]. Indeed, individuals with IBD exhibit relatively lower levels of SQOR, a key enzyme responsible for H₂S detoxification [93]. Second, alterations in the gut microbiota significantly influence exogenous H₂S production. In IBD, SRB—a group of flagellated gut microbes—produce hydrogen sulfide (H₂S) as a terminal metabolite [92, 94]. The H₂S biosynthesis pathway in SRB proceeds as follows: sulfate, a highly stable molecule, is first converted to sulfite (SO₃^2^⁻) through the actions of ATP sulfurylase and APS reductase. Sulfite is then further reduced by dissimilatory sulfite reductase (dSiR), resulting in the formation of H₂S [95, 96]. Therefore, excessive proliferation of gut microbes such as SRB can lead to the overproduction of H₂S within the intestinal lumen. Elevated luminal H₂S levels inhibit cytochrome c oxidase in colonocyte mitochondria, thereby impairing ATP synthesis. This mitochondrial dysfunction may result in decreased cellular function, potentially leading to apoptosis or functional damage to the intestinal epithelium. Additionally, excess H₂S can reduce the disulfide bonds (–S–S–) in mucin proteins, compromising the structural stability of the mucus layer and disrupting the physical barrier of the intestinal epithelium [75, 95, 97]. This dual insult—mitochondrial dysfunction and mucus barrier weakening—facilitates the translocation of pathogens and endotoxins, further exacerbating mucosal inflammation and thereby perpetuating a pathophysiological vicious cycle [78, 98]. Indeed, patients with IBD exhibit increased SRB activity and H₂S levels, along with decreased levels of beneficial microbial metabolites, compared to healthy individuals [86, 99, 100]. Notably, patients in the active phase of the disease exhibit elevated levels of SRB relative to those in remission [101–103]. This increase, within the context of an inflammatory environment, further promotes SRB proliferation while simultaneously impairing H₂S detoxification capacity, thereby exacerbating mucosal injury in the colon [94, 104]. These findings suggest that SRB overgrowth and exogenous H₂S accumulation are closely linked to IBD pathogenesis and are strongly correlated with disease severity. Third, the dysregulation of H₂S metabolism also represents a critical contributing factor. In mammals, the primary enzymes responsible for endogenous H₂S biosynthesis include cystathionine-β-synthase (CBS), cystathionine-γ-lyase (CSE), and 3-mercaptopyruvate sulfurtransferase (3-MST) [87, 105]. Endogenously produced H₂S exerts various physiological effects, including anti-inflammatory activity, promotion of tissue repair, maintenance of intestinal barrier integrity, suppression of oxidative stress, and protection against inflammation-induced tissue damage [75, 106]. However, patients with IBD frequently exhibit reduced expression of endogenous H₂S-producing enzymes, likely due to impaired vitamin B6 absorption, gut microbiota dysbiosis, and immune dysfunction [107–109]. This reduction compromises the anti-inflammatory defenses of the intestinal mucosa, weakens epithelial barrier integrity, and increases oxidative stress, thereby exacerbating IBD pathogenesis [110–112]. Supporting this, studies have shown that genetic inhibition of CBS in mice increases vascular permeability and promotes leukocyte adhesion to endothelial cells, thereby enhancing inflammatory signaling [77, 113]. Immunohistochemical analyses of intestinal epithelial cells from adult IBD patients have also revealed reduced expression of all three key H₂S-producing enzymes [85, 93]. In vitamin B6-deficient mice, CSE expression is diminished, and pharmacological inhibition of CSE has been shown to aggravate colitis and delay tissue repair [79]. Similarly, 3-MST-deficient mice exhibit elevated levels of proinflammatory cytokines and more severe colitis, consistent with the marked reduction of 3-MST expression observed in the colonic mucosa of IBD patients [93, 112]. These findings underscore the protective role of endogenous H₂S in maintaining intestinal homeostasis and indicate that disruption of its biosynthesis contributes to disease progression. As a therapeutic strategy to restore this balance, the delivery of H₂S donors to inflamed colonic regions has shown significant therapeutic potential in various preclinical studies [25, 30, 114]. According to several reports, exogenous H₂S donors promote mucosal healing by restoring microbial balance, preserving the structure of the mucus-biofilm barrier, and alleviating chronic inflammation [26, 75, 115].

In conclusion, given the three major factors contributing to hydrogen sulfide (H₂S) imbalance, the development of a colon-targeted delivery system capable of precisely maintaining H₂S levels within the therapeutic range is crucial for effective IBD treatment, as it can enhance the anti-inflammatory and cytoprotective effects of H₂S while minimizing its potential toxicity. To this end, recent studies have increasingly reported on the potential of nanoparticle-based oral delivery systems for IBD treatment (Table 1). Among various therapeutic agents, mesalazine (5-ASA) remains one of the most widely used drugs for the treatment of IBD [116, 117]. Soumayya Aib et al. developed a pH-sensitive liposomal system (MZ-CM co-loaded LS) that co-encapsulates low-dose mesalazine (MZ) and curcumin (CM). The formulation was coated with Eudragit S100 to enable colon-targeted drug delivery, and it effectively alleviated ulcerative colitis (UC) through the synergistic anti-inflammatory and antioxidant effects of both drugs. Compared to single-drug formulations, MZ-CM LS demonstrated superior therapeutic efficacy by enabling sustained and localized drug release in the colon [118]. Anas Ahmad et al. designed an oral formulation in which gelatin nanoparticles loaded with 5-ASA were coated with Eudragit S100. In vitro release studies demonstrated minimal drug release (1.3%) at gastric pH (2.0), while a significantly enhanced release (∼82%) was observed at colonic pH (7.4) after 72 h. In a DSS-induced colitis mouse model, this formulation exhibited superior therapeutic outcomes compared to free 5-ASA [119]. Lei Qiu et al. developed a targeted delivery system in which a nanodietary fiber conjugate (NDF-1-Pro/5-ASA), formed by linking 5-ASA to BSA and IL-1β antibodies, was co-encapsulated with Bifidobacterium into alginate-based microparticles (NDF-M) and coated with polylysine (PLL). This system released over 70% of 5-ASA within 6–16 h at pH 7.0 and demonstrated therapeutic efficacy in a chronic colitis mouse model by significantly reducing the expression of IL-1β and TNF-α, as well as inflammatory markers such as MPO, H₂O₂, and MDA [120]. Min Wang et al. developed an oral solid lipid nanoparticle (SLNP) system (F5) containing mesalazine (MES), coated with sodium alginate. In a TNBS-induced colitis model, MES-SLNPs showed notable therapeutic efficacy compared to MES suspension, as evidenced by improved body weight recovery, restoration of colon length, and reduced inflammatory cell infiltration and tissue damage [121]. Solmaz Mahami et al. designed an oral delivery system in which mesalazine (5-ASA) was encapsulated into chitosan nanoparticles (CSNPs) cross-linked with hydroxypropyl methylcellulose phthalate (HPMCP). The formulation suppressed drug release in simulated gastric fluid (SGF) and enabled efficient release in simulated intestinal fluid (SIF). In an AA-induced colitis model, the system demonstrated anti-inflammatory effects [122]. Nan Wang et al. developed pH-sensitive core–shell nanoparticles (ES₁CS₅SA₅@5-ASANCs) by sequentially coating 5-ASA nanocrystals with chitosan (CS), sodium alginate (SA), and Eudragit S100 (ES). In vitro studies showed that the formulation suppressed premature drug release under acidic conditions and sustained release in colonic environments. Biodistribution and mucus penetration studies further confirmed strong colonic accumulation and excellent mucus permeability [123]. Rupinderjeet Kaur et al. developed a synbiotic-based colon-targeted drug delivery system in which mesalazine was encapsulated into microspheres composed of guar gum and xanthan gum (both serving as prebiotics), co-administered with probiotics, including Lactobacillus and Bifidobacterium. In a rat model, this formulation significantly improved outcomes in terms of fecal parameters, body weight gain, and histopathological recovery [124]. Souvik Mohanta et al. proposed a colon-targeted strategy that combines mesalazine (MES) with modified apple polysaccharide (MAP) into mini-tablets, co-administered with probiotics. The MES mini-tablets were formulated using guar gum cores coated with Eudragit S100 and guar gum. In an acetic acid-induced UC rat model, the combination treatment exhibited the highest therapeutic efficacy compared to control groups [125].

**Table 1:** Oral mesalazine formulations for the treatment of IBD

Name of NPs or chemical	Types of coating agent	Size	PDI	Type of used drug or materials	References
MZ-CM co-loaded LS	Eudragit S100	212.8 ± 6.2 nm	0.143	Mesalazine and curcumin	[118]
EUD S100-coated 5-ASA loaded NPs	Eudragit S100	243.8 ± 68.7 nm	0.2	5-ASA	[119]
NDF-M	polylysine	100–500 nm	N/A	5-ASA and Bac	[120]
MES-SLNPs	sodium alginate	217.4 ± 0.6 nm	0.160 ± 0.03	Mesalazine	[121]
5-ASA/HPMCP/ CSNPs	HPMCP	281.2 ± 25.80	0.22 ± 0.07	5-ASA and berberine	[122]
ES₁CS₅SA₅@5-ASANCs	Eudragit S100/Chitosan/ Sodium Alginate	352 ± 2 nm	0.25 ± 0.01	5-ASA	[123]
Mesalamine loaded polysaccharide microspheres	Guar gum and xanthan gum	291.02 μm	0.421	Mesalazine	[124]
MES-MAP mini tablets	Eudragit S100 and Guar gum	N/A	N/A	Mesalazine	[125]

MZ: mesalazine, CM: curcumin, LS: pH-sensitive liposomes, Eud: Eudragit S100, 5-ASA: 5-aminosalicylic acid, NPs: nanopaticles, NDF: nanoscale dietary fibers, M: microspheres, NDF-M: NDF-1-pro/5-ASA microsphere, Bac: bifidobacterium, MES: mesalazine, SLNPs: solid lipid nanoparticles, MES-SLNPs: Mesalamine loaded SLNPs, HPMCP: hydroxypropyl methylcellulose phthalate, CSNPs: chitosan nanoparticles, ES: Eudragit S100, CS: chitosan, SA: sodium alginate, ASANCs: 5-Aminosalicylic acid nanocrystals, ES₁CS₅SA₅@5-ASANCs: pH sensitive 5-aminosalicylic acid core–shell nanoparticles, MES: mesalazine, MAP: Modified Apple Polysaccharide, EAC: Eudragit S100-aminoclay, EAC-IFX-L: Eudragit S100-aminoclay-liposome-coated IFX, IFX: infliximab, AC: aminoclay, AC-IFX-L: aminoclay-liposome-coated IFX, Ch: chitosan, Ch-Mn₃O₄ NPs: Chitosan-capped Mn₃O₄ nanoparticles. PDI: polydispersity index, N/A: Not applicable

Subsequently, the selection of coating materials remains a critical design factor in the development of orally administered nanoparticles for colon-targeted delivery systems [126, 127]. Among the various colon-specific drug delivery strategies, Eudragit S100 is an anionic copolymer of methacrylic acid and methyl methacrylate widely used as an enteric coating and in controlled-release formulations. It is commonly applied to enable the oral delivery of nanoparticles, including liposomes and lipid nanoparticles (LNPs). Under acidic conditions, its carboxylic acid groups remain protonated, rendering the polymer non-ionized and poorly soluble; this hydrophobic barrier protects encapsulated nanoparticles from acidic environment and enzymatic degradation. In contrast, at pH ≥ 7, deprotonation increases hydrophilicity, the coating dissolves (or swells and erodes), and the nanoparticles are rapidly released [59, 128, 129]. Additional polymers, including HPMCP, poly-L-lysine, chitosan, sodium alginate, guar gum, and xanthan gum are employed based on their distinct physicochemical properties and biocompatibility. HPMCP, like Eudragit S100, is a pH-sensitive polymer that enables delayed drug release across the small intestine and colon [130]. Poly-L-lysine and chitosan are used to improve mucoadhesion, increase epithelial permeability, or stabilize the mucosal surface [131, 132]. Sodium alginate, guar gum, and xanthan gum are commonly used to achieve sustained and controlled release profiles [133–135]. However, assuming uniform colonic pH and transit profiles oversimplifies the physiological diversity observed in IBD patients. Clinical studies demonstrate that active inflammation often lowers colonic pH relative to healthy individuals, potentially delaying the dissolution of Eudragit S100 coatings [136–138]. Moreover, gastrointestinal transit times vary widely in IBD: some patients experience accelerated motility due to diarrhea, whereas others show delayed passage owing to strictures or dysmotility [139, 140]. This heterogeneity in luminal pH and transit kinetics may influence the site and rate of drug release, thereby affecting therapeutic performance [138–140]. While our formulation is expected to remain stable under acidic conditions and release its payload upon encountering neutral to slightly alkaline environments, the possibility of delayed or incomplete release in subsets of patients should be acknowledged. Future adaptations could therefore incorporate complementary triggers to enhance formulation robustness and ensure consistent therapeutic outcomes across diverse patient subgroups.

Importantly, as summarized in Table 2, most oral colon-targeted nanoparticle systems reported to date have employed multi-layered or composite coating strategies, hybrid designs incorporating microbiota-responsive polysaccharides, or chemically modified polymers to achieve enhanced therapeutic efficacy [141]. Representative examples include hyaluronic acid–stabilized zein/caseinate complexes, pectin–chitosan hybrids, and carboxymethyl chitosan micelles. In contrast, our formulation demonstrated that a single enteric coating with Eudragit S100 alone was sufficient to provide robust gastric protection and enable efficient colonic release leading to a statistically significant therapeutic improvement, as evidenced by increased colon length in DSS-induced colitis (p < 0.001). Importantly, this study reports the first oral nano-formulation of a hydrogen sulfide donor (GYY4137), thereby establishing a novel therapeutic paradigm that extends beyond the conventional delivery of anti-inflammatory agents. Nevertheless, although the single Eudragit S100 coating exhibited considerable therapeutic potential in Oral H₂S donor delivery, inter-individual variability in pathophysiological conditions may still affect release profiles. Future research should consider composite strategies, such as microbiota-responsive or time-dependent coatings, to further refine pH-dependent release control and thereby improve formulation stability and strengthen the translational potential of this platform.

**Table 2:** Nanoparticles and drugs with oral delivery for IBD treatment

**Name of NPs or chemical**	**Targeting system**	**Coating agents**	**Degradation strategy**	**Size**	**PDI**	**Type of used drug**	**Therapeutic effect** **(colon length, vs DSS)**	**References**
ISL@NPs	Zein/Cas complex NPs	-	Microbiota-responsive enzymatic + pH-triggered degradation	137.32 ± 2.54 nm	0.13 ± 0.01	ISL	Significant improvement (p < 0.05)	[64]
CZNH NPs	Zein/Cas complex NPs	Hyaluronic acid	Microbiota-responsive enzymatic + pH-triggered degradation	250 nm	0.13 ± 0.02	Curcumin	Significant improvement (p < 0.05)	[142]
Tof@BSA-Chs-CP NPs	BSA NPs	Pectin + chitosan	Microbiota-responsive enzymatic + pH-triggered degradation	300 nm	0.065 ± 0.028	Tof	Significant improvement (p < 0.001)	[143]
CMC-AXT-NPs	Carboxymethyl chitosan nanomicelles	Carboxymethyl chitosan	Microbiota-responsive enzymatic + pH-triggered degradation	34.5 nm	0.33	AXT	Significant improvement (p < 0.001)	[144]
Oral H_2_S lipo	Liposome NPs	Eudragit S100	pH-triggered degradation	194.9 nm	0.157	H_2_S donor (GYY4137)	Significant improvement (p < 0.001)	This study

NPs: nanoparticles, ISL: isoliquiritigenin, Cas: caseinate, CZNH: curcumin loaded biopolymeric nanocomposite, Tof: Tofacitinib, BSA: bovine serum albumin, Chs: chondroitin sulfate, CP: chitosan/pectin shell, CMC-AXT-NPs: carboxymethyl chitosan-modified astaxanthin-loaded nanoparticles, AXT: astaxanthin, H_2_S: hydrogen sulfide. PDI: polydispersity index, N/A: Not applicable

## Conclusions

In this study, we developed a pH-responsive, colon-targeted liposomal system for oral drug delivery. The formulation, termed Oral H₂S lipo, consists of GYY4137 (an H₂S donor)-loaded liposomes coated with Eudragit S100. We assessed its physicochemical and biological performance, demonstrating that the Eudragit S100 coating exhibits clear pH-sensitive behavior, aggregating under acidic conditions (pH 3) and dissolving at neutral pH (pH 7). Oral H₂S lipo showed high loading efficiency of GYY4137 and maintained excellent stability across a range of pH conditions—including acidic (pH 3), mildly acidic (pH 4), and neutral (pH 7) environments. In vitro cytotoxicity assays using HFF and RAW 264.7 cell lines confirmed its biocompatibility, and in vivo imaging in a colitis mouse model revealed enhanced colon-targeting efficiency with minimal off-target accumulation. Oral H₂S lipo alleviated body weight loss and colon shortening in a DSS-induced colitis mouse model, and effectively mitigated histological inflammation by reducing mucosal damage and immune cell infiltration, confirming its therapeutic benefits. These findings indicate that Oral H₂S lipo may function as a viable colon-targeted oral drug delivery system. Our study is the first to apply GYY4137 in an oral formulation, thereby offering a promising therapeutic strategy and highlighting the potential of Oral H₂S lipo as an innovative and effective platform for IBD treatment.

## Supplementary Information

Additional file1

## Data Availability

No datasets were generated or analysed during the current study.
