# Supplementary material for: pH-responsive Oral liposomal delivery of hydrogen sulfide donor GYY4137 enables colon-targeted therapy for inflammatory bowel disease
Source: J Nanobiotechnology. 2025 Nov 11;23:712. doi: 10.1186/s12951-025-03753-9 (PMC12606812; doi:10.1186/s12951-025-03753-9)
Supplement: Supplementary file 1 — Additional file1 [file 12951_2025_3753_MOESM1_ESM.pptx]

## Slide 1
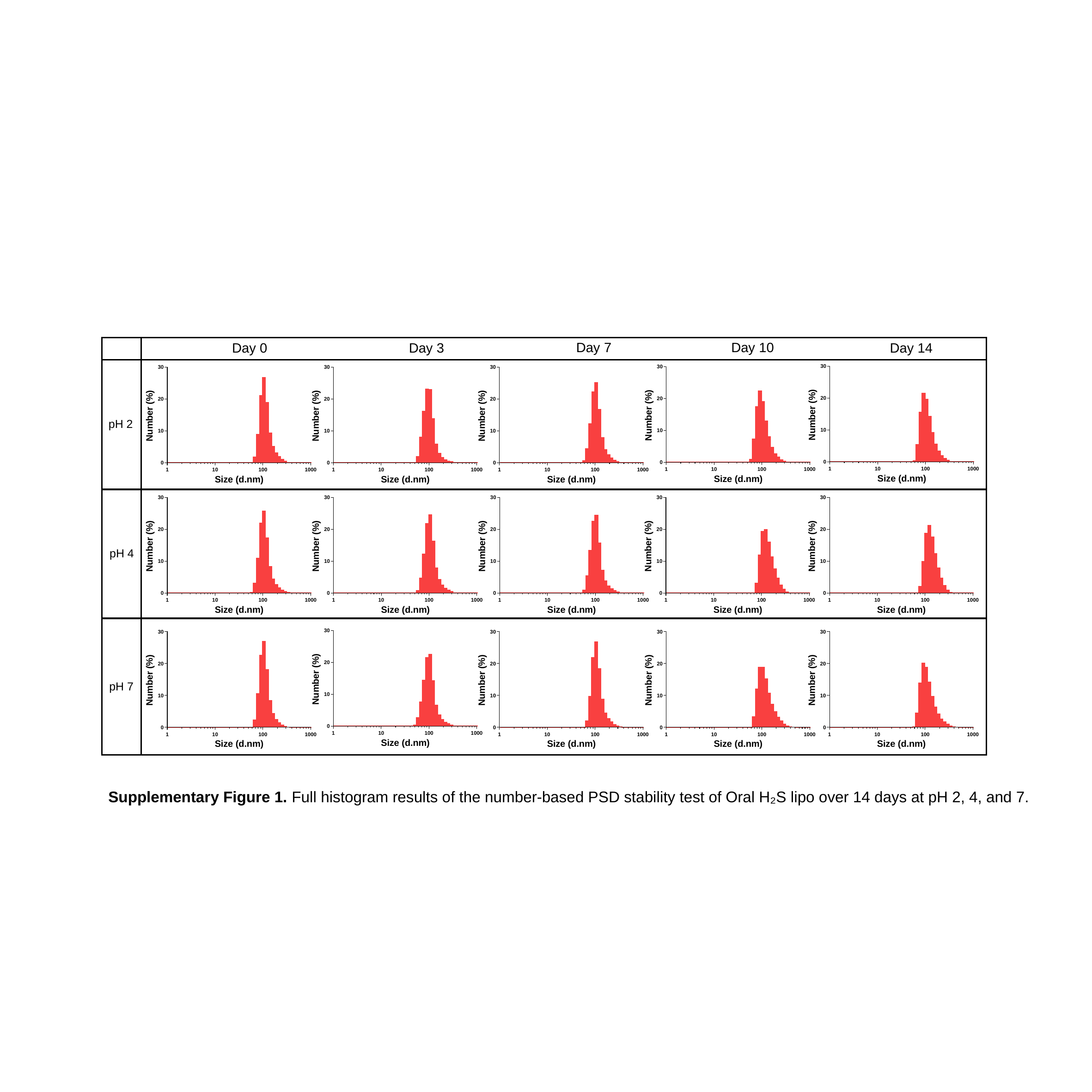

Day 10
Day 7
Day 3
Day 14
Day 0
pH 2
pH 4
pH 7
Supplementary Figure 1. Full histogram results of the number-based PSD stability test of Oral H₂S lipo over 14 days at pH 2, 4, and 7.

## Slide 2
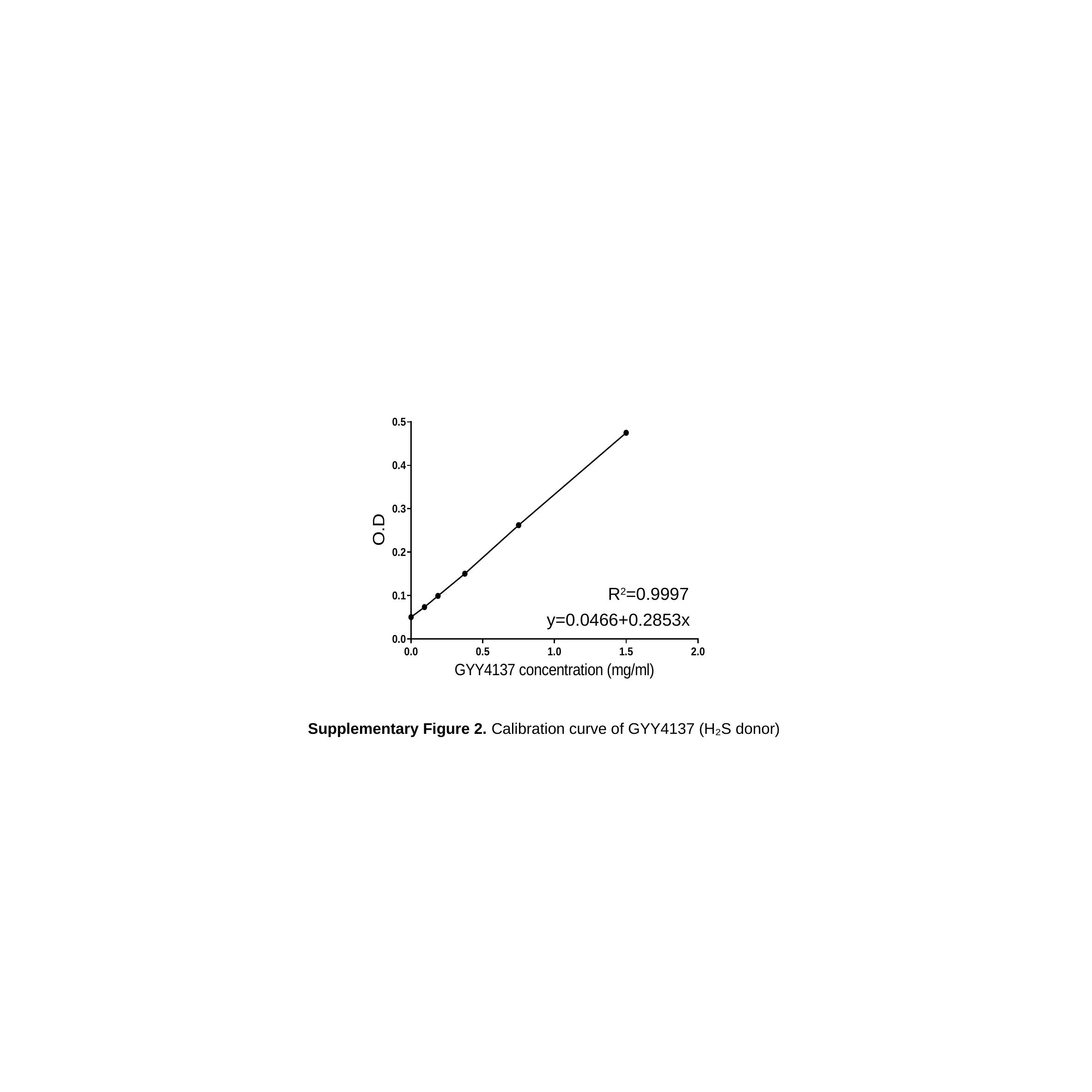

R2=0.9997
y=0.0466+0.2853x
Supplementary Figure 2. Calibration curve of GYY4137 (H₂S donor)

## Slide 3
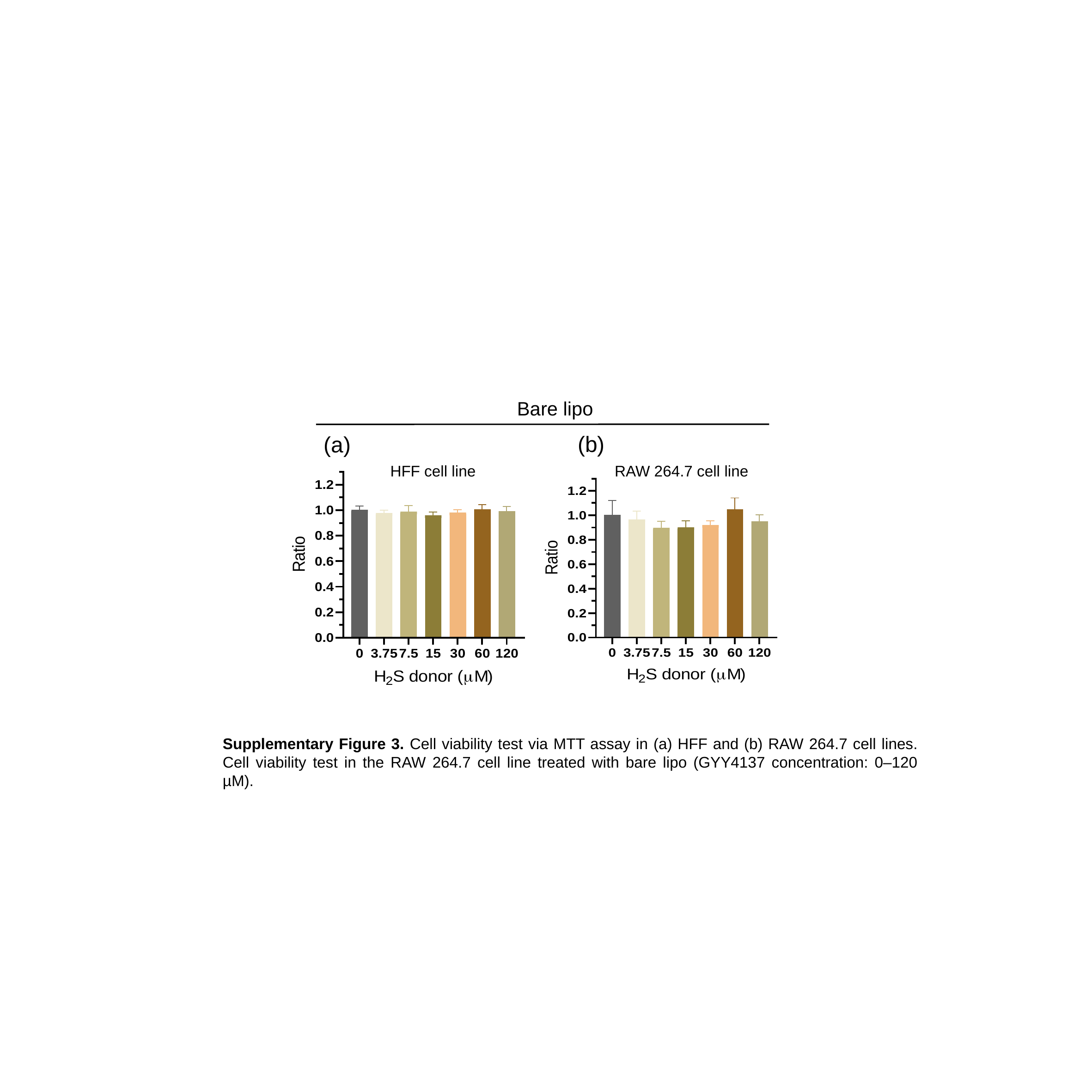

Bare lipo
(b)
(a)
HFF cell line
RAW 264.7 cell line
Supplementary Figure 3. Cell viability test via MTT assay in (a) HFF and (b) RAW 264.7 cell lines. Cell viability test in the RAW 264.7 cell line treated with bare lipo (GYY4137 concentration: 0–120 µM).

## Slide 4
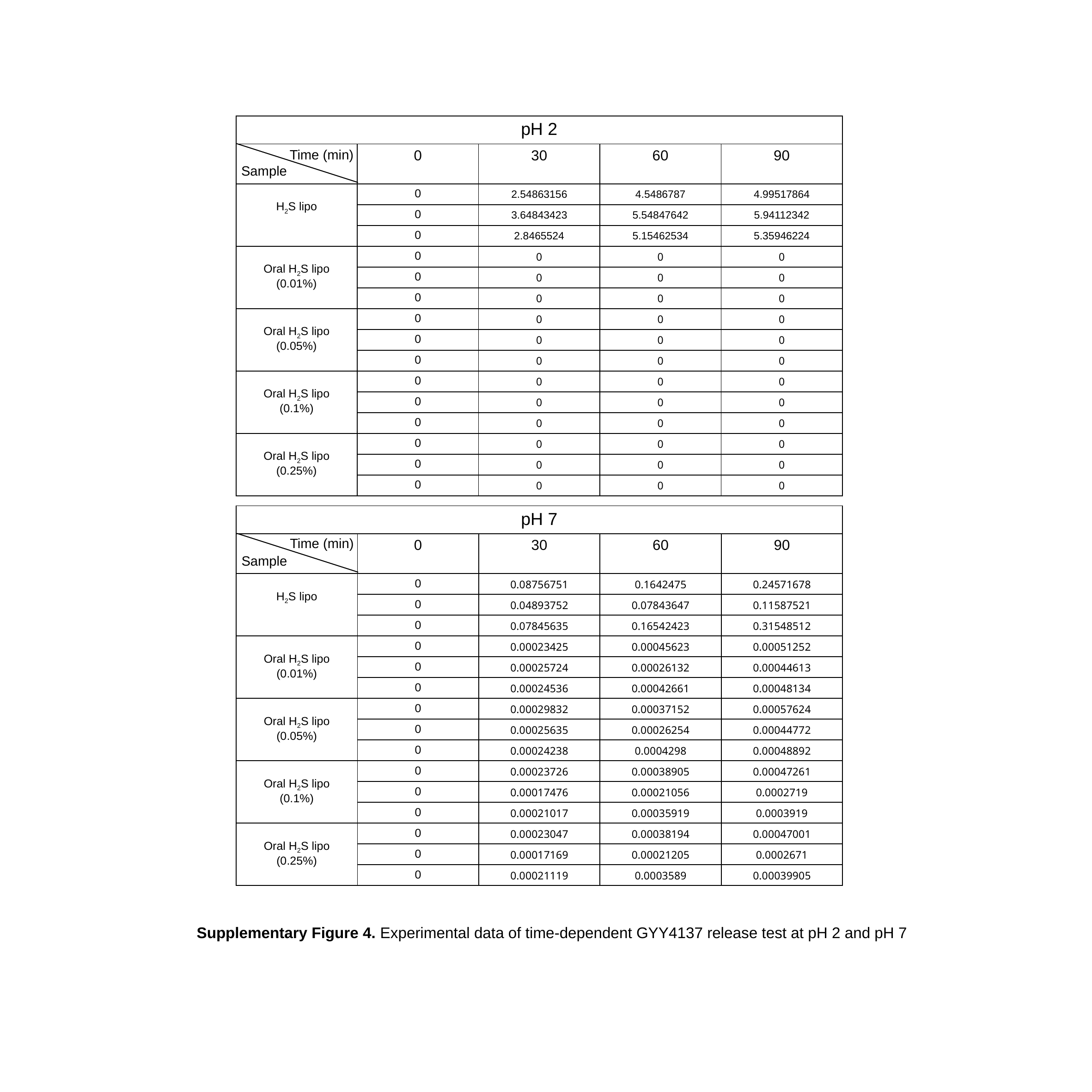

| pH 2 | | | | |
| --- | --- | --- | --- | --- |
| | 0 | 30 | 60 | 90 |
| H2S lipo | 0 | 2.54863156 | 4.5486787 | 4.99517864 |
| | 0 | 3.64843423 | 5.54847642 | 5.94112342 |
| | 0 | 2.8465524 | 5.15462534 | 5.35946224 |
| Oral H2S lipo (0.01%) | 0 | 0 | 0 | 0 |
| | 0 | 0 | 0 | 0 |
| | 0 | 0 | 0 | 0 |
| Oral H2S lipo (0.05%) | 0 | 0 | 0 | 0 |
| | 0 | 0 | 0 | 0 |
| | 0 | 0 | 0 | 0 |
| Oral H2S lipo (0.1%) | 0 | 0 | 0 | 0 |
| | 0 | 0 | 0 | 0 |
| | 0 | 0 | 0 | 0 |
| Oral H2S lipo (0.25%) | 0 | 0 | 0 | 0 |
| | 0 | 0 | 0 | 0 |
| | 0 | 0 | 0 | 0 |
Time (min)
Sample
| pH 7 | | | | |
| --- | --- | --- | --- | --- |
| | 0 | 30 | 60 | 90 |
| H2S lipo | 0 | 0.08756751 | 0.1642475 | 0.24571678 |
| | 0 | 0.04893752 | 0.07843647 | 0.11587521 |
| | 0 | 0.07845635 | 0.16542423 | 0.31548512 |
| Oral H2S lipo (0.01%) | 0 | 0.00023425 | 0.00045623 | 0.00051252 |
| | 0 | 0.00025724 | 0.00026132 | 0.00044613 |
| | 0 | 0.00024536 | 0.00042661 | 0.00048134 |
| Oral H2S lipo (0.05%) | 0 | 0.00029832 | 0.00037152 | 0.00057624 |
| | 0 | 0.00025635 | 0.00026254 | 0.00044772 |
| | 0 | 0.00024238 | 0.0004298 | 0.00048892 |
| Oral H2S lipo (0.1%) | 0 | 0.00023726 | 0.00038905 | 0.00047261 |
| | 0 | 0.00017476 | 0.00021056 | 0.0002719 |
| | 0 | 0.00021017 | 0.00035919 | 0.0003919 |
| Oral H2S lipo (0.25%) | 0 | 0.00023047 | 0.00038194 | 0.00047001 |
| | 0 | 0.00017169 | 0.00021205 | 0.0002671 |
| | 0 | 0.00021119 | 0.0003589 | 0.00039905 |
Time (min)
Sample
Supplementary Figure 4. Experimental data of time-dependent GYY4137 release test at pH 2 and pH 7

## Slide 5
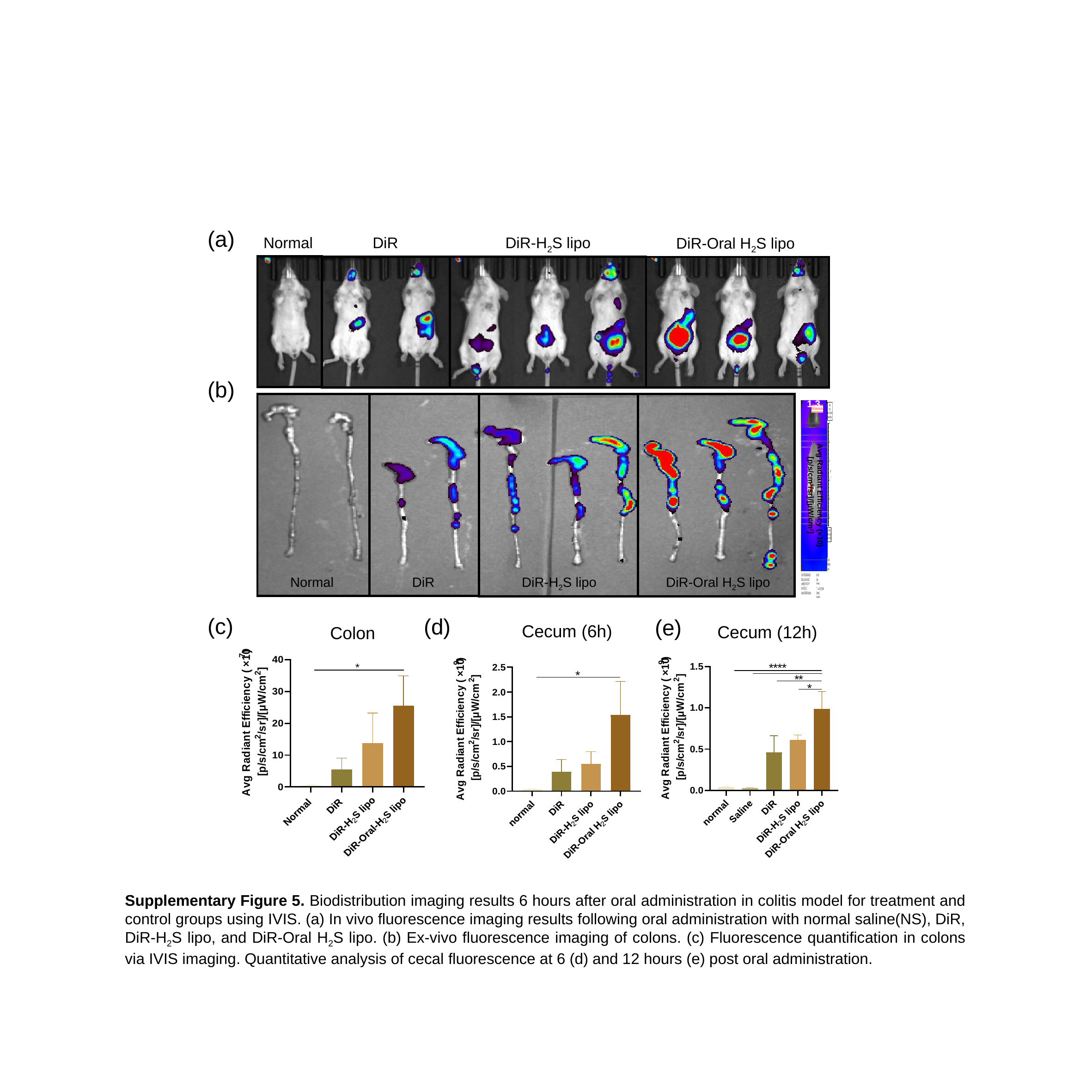

(a)
Normal
DiR
DiR-H2S lipo
DiR-Oral H2S lipo
Normal
DiR
DiR-H2S lipo
DiR-Oral H2S lipo
1.3
Avg Radiant Efficiency (×10)
 [p/s/cm2/sr]/[​μW/cm2]
0.2
(b)
(c)
(d)
(e)
Cecum (6h)
Cecum (12h)
Colon
Supplementary Figure 5. Biodistribution imaging results 6 hours after oral administration in colitis model for treatment and control groups using IVIS. (a) In vivo fluorescence imaging results following oral administration with normal saline(NS), DiR, DiR-H2S lipo, and DiR-Oral H2S lipo. (b) Ex-vivo fluorescence imaging of colons. (c) Fluorescence quantification in colons via IVIS imaging. Quantitative analysis of cecal fluorescence at 6 (d) and 12 hours (e) post oral administration.

## Slide 6
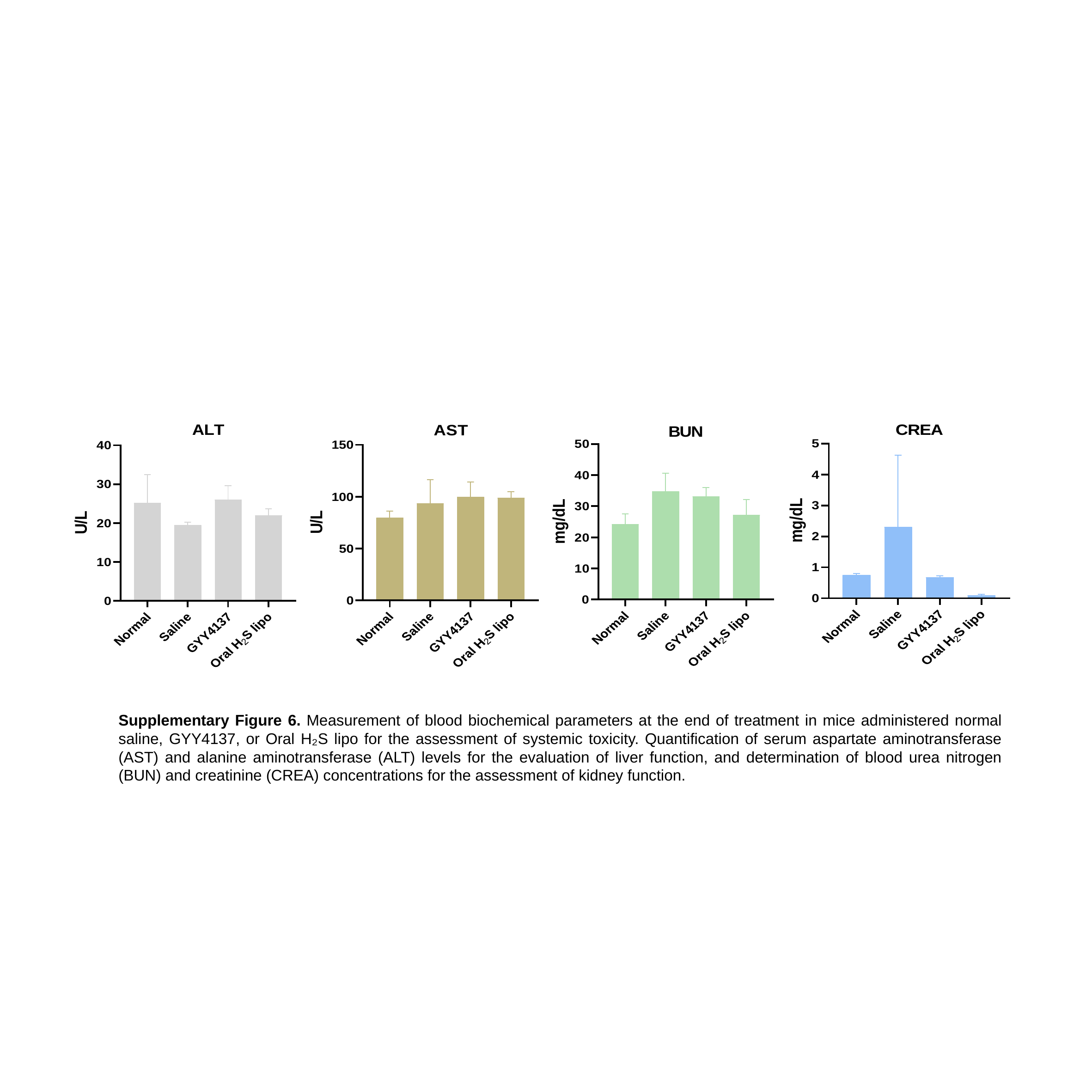

Supplementary Figure 6. Measurement of blood biochemical parameters at the end of treatment in mice administered normal saline, GYY4137, or Oral H₂S lipo for the assessment of systemic toxicity. Quantification of serum aspartate aminotransferase (AST) and alanine aminotransferase (ALT) levels for the evaluation of liver function, and determination of blood urea nitrogen (BUN) and creatinine (CREA) concentrations for the assessment of kidney function.

## Slide 7
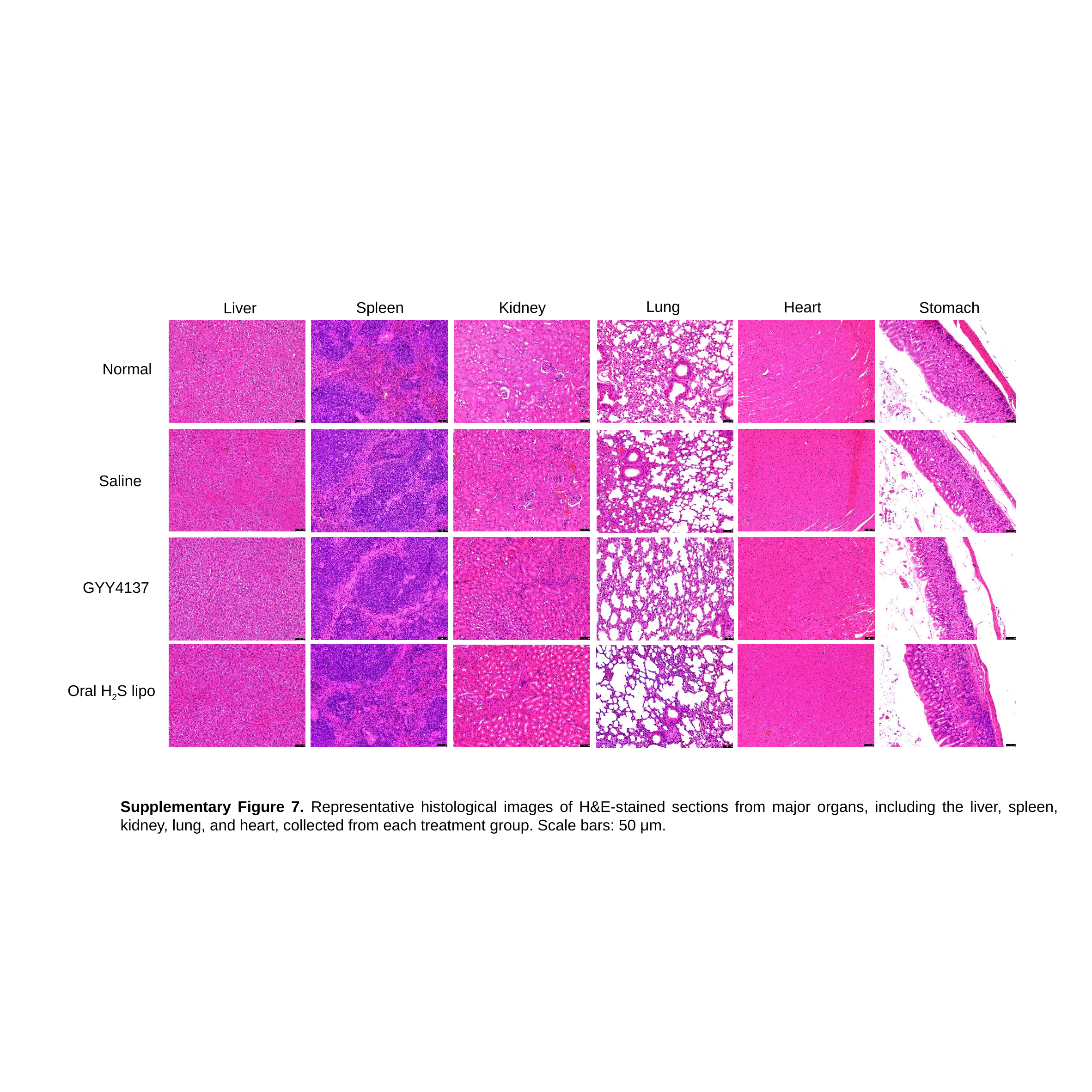

Lung
Heart
Spleen
Stomach
Kidney
Liver
Normal
Saline
GYY4137
Oral H2S lipo
Supplementary Figure 7. Representative histological images of H&E-stained sections from major organs, including the liver, spleen, kidney, lung, and heart, collected from each treatment group. Scale bars: 50 μm.
